# Ruthenium Complexes Containing Pyridinyl-Derived Ligands as FLP Catalysts

**DOI:** 10.1021/acs.inorgchem.6c00833

**Published:** 2026-05-14

**Authors:** Alejandro Grasa, Hannah Middlebrook, Réka Anna Józsa, Vincenzo Passarelli, Fernando Viguri, Ricardo Rodríguez, Pilar Lamata

**Affiliations:** 201458 Instituto de Síntesis Química y Catálisis Homogénea (ISQCH), CSIC-Universidad de Zaragoza, Departamento de Química Inorgánica, Pedro Cerbuna 12, Zaragoza 50009, Spain

## Abstract

We report the synthesis of ruthenium-based frustrated Lewis pair (FLP) complexes of the general formula [(Mes)­Ru­(κ^3^ *N*,*N*′,*N″*-**L**)]­[SbF_6_], where Mes denotes η^6^-1,3,5-C_6_H_3_Me_3_ and **L** corresponds to two different ligands: **L1** (*N*,*N*′-bis­(2,6-diisopropylphenyl)-*N″*-(2-pyridinylmethyl)­guanidinate) in complex **1** and **L2** (*N*-2-pyridinyl-*N*-(2-pyridinylmethyl)­amidate) in complex **4**. These complexes are obtained from the dimer [{(Mes)­RuCl}_2_(μ-Cl)_2_] in combination with **HL1** or **HL2** under chloride abstraction and deprotonation conditions. Complexes **1** and **4** undergo reversible heterolytic activation of H_2_ to form the corresponding hydrido species [(Mes)­RuH­(κ^2^ *N*,*N*′-**HL**)]­[SbF_6_] (**HL** = **HL1** for complex **6**; **HL2** for complex **7**). The formation of these hydrido complexes is observed in both the hydrodechlorination of chlorinated substrates under H_2_ and the base-free dehydrogenation of formic acid, highlighting the reversibility of hydrogen activation within this TMFLP framework. Finally, complexes **1** and **4** act as transition-metal frustrated Lewis pair (TMFLP) hydrogenation catalysts and catalyze the hydrogenation reaction of C=C bonds in acrylates (even in water for **1**), as well as the C=O bond of 2,2,2-trifluoroacetophenone, and the C=N bond of *N*-benzylideneaniline.

## Graphical abstract

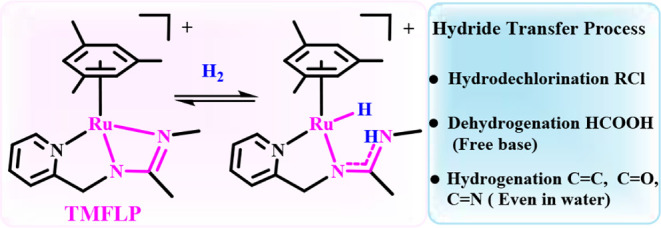

## Introduction

Frustrated Lewis pairs (FLPs) are combinations of Lewis acids and bases capable of activating small molecules and catalyzing their transformation, a reactivity paradigm known as FLP chemistry. This behavior arises when acid–base interaction is sterically or electronically inhibited, thus preventing the formation of a stable Lewis adduct and allowing both components to remain reactive toward an external substrate.^1^ As a result, the acid and base can cooperatively bind and activate small molecules in a concerted manner.

Although early examples of FLP chemistry were primarily associated with steric hindrance, it has since been demonstrated that this reactivity is considerably more general. In particular, FLP behavior can emerge whenever an equilibrium exists between a classical Lewis adduct and its dissociated form, that is, the latter is thermally accessible.^2^ The concept of frustrated Lewis pairs was formally introduced in 2007,^3^ following the discovery that Mes_2_P­(C_6_F_4_)­B­(C_6_F_5_)_2_ undergoes reversible activation of dihydrogen under mild conditions.^4^ Initial FLP systems predominantly involved boron-based Lewis acids paired with phosphorus- or nitrogen-based Lewis bases. Subsequent developments have significantly expanded the scope of FLP chemistry to include a broad range of donor elements from groups 14–16, such as C, O, Se, and Te, and acceptor elements from groups 13–15, including Al, Ga, In, C, Si, and Sn.^5^ This diversification has enabled the activation of numerous small molecules, e.g., CO_2_, CO, SO_2_, N_2_O, and NO, as well as unsaturated organic substrates, such as olefins and alkynes, through cooperative mechanisms.^5,6^

One of the most significant impacts of FLP chemistry lies in its application to catalysis. In this context, the incorporation of transition metals into FLP frameworks has given rise to transition-metal frustrated Lewis pairs (TMFLPs),^7^ which offer enhanced structural diversity (different oxidation states and coordination modes of the metal, and a wide variety in electronic and steric characteristics of the ligand), and access to fundamental catalytic transformations (a result from the presence of partly occupied *d* orbitals in transition metals). The combination of the properties of transition-metal complexes with the concept of frustration, in which the FLP is more reactive than its segregated components, can lead to the development of catalytic systems that enhance the capabilities of current catalysts. The first TMFLP systems were reported by the groups of Wass^7,8^ and Erker^9^ using Zr/phosphine-based pairs. Complementing these systems, Meyer and coworkers described the first example of a Ru­(II) complex acting as a Lewis base in an FLP framework.^10^ More recently, Campos and coworkers reported the first FLP system composed exclusively of transition metals (TMOFLPs), featuring Au­(I) and Pt(0) complexes as the Lewis acidic and basic components, respectively.^11^ Extensions into the f-block have also been reported by the Liddle^12^ and Arnold^13^ groups, employing metals such as U­(III) and Nd­(III).

Our recent studies have demonstrated that rhodium,^14a,14d^ iridium,^14b,14c^ ruthenium,^14e,14g^ and osmium^14f,14g^ complexes bearing phosphano- and pyridinyl-guanidinate ligands act as TMFLPs capable of reversibly activating dihydrogen. In particular, the ruthenium and osmium systems exhibit activity in the hydrogenation of substrates containing C=C, C=O, and C=N bonds.^14g^ However, the catalytic performance of complexes featuring pyridinyl-guanidinate ligands can be compromised by ortho-metalation of the *p*-tolyl substituent^14g^ and by the lability of the *p*-cymene ligand. To address these limitations and develop more robust TMFLP catalysts, we have designed two new (Mes)­Ru­(**L**) systems, where **L** is either a pyridinyl-guanidinate ligand bearing isopropyl groups at the *ortho* positions of the phenyl rings (**L1**) or a pyridinyl-amidate ligand (**L2**). These ligands retain the essential electronic and steric features required for effective TMFLP formation.

TMFLP catalysis is an example of cooperative catalysis that involves the concerted action of segregated Lewis acid and basic sites on a substrate molecule. In the present systems, the acid will be ruthenium and the base will be a nitrogen atom from the ligands **L1** and **L2**.^1a^ Herein, we report: (i) the synthesis and characterization of the new FLP complexes [(Mes)­Ru­(κ^3^ *N*,*N*′,*N*″-**L**)]­[SbF_6_] (**L** = **L1** (**1**), **L2** (**4**)); (ii) their reactivity toward molecular hydrogen; (iii) their reactivity toward chlorinated substrates under H_2_; (iv) the application of complexes **1** and **4** as catalysts for the base-free dehydrogenation of formic acid; and (v) their catalytic activity in the hydrogenation of C=C (acrylates, including reactions in water with complex **1**), C=O (2,2,2-trifluoroacetophenone) and C=N (*N*-benzylideneaniline) bonds.

## Results and Discussion

### Synthesis of the Ligands

Ligand *N*,*N*′-bis­(2,6-diisopropylphenyl)-*N″*-(2-pyridinylmethyl)­guanidine (**HL1**) was prepared in 94% yield by reaction of 2-(2-aminoethyl)­pyridine with bis­(2,6-diisopropylphenyl)­carbodiimide in dry THF following reported procedures^15^ (Scheme 1a). **HL1** was characterized in the solid state by single-crystal X-ray diffraction (Figure S1, SI). Ligand *N*-2-pyridinyl-*N*-(2-pyridinylmethyl)­amine (**HL2**) was obtained in two steps, condensation of 2-aminopyridine with 2-pyridinecarboxaldehyde in toluene, followed by reduction of the resulting imine with NaBH_4_ in ethanol (overall yield 47%), according to the literature (Scheme 1b).^16^

**Scheme 1:**
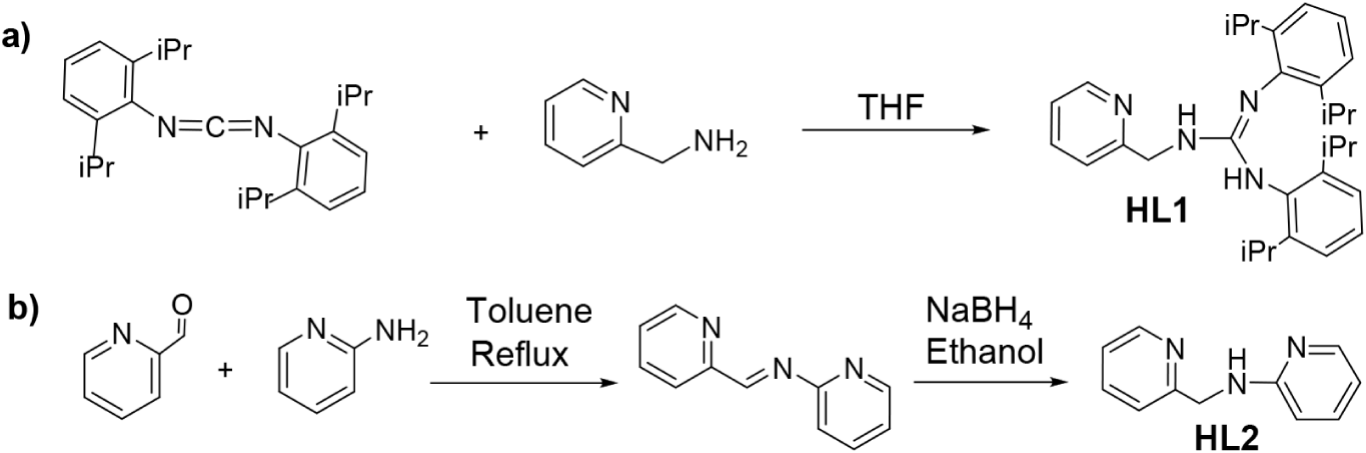
Preparation of **HL1** (a) and **HL2** (b) Ligands

### Synthesis of the Complex [(Mes)­Ru­(κ^3^ *N,N*′,*N″*-L1)]­[SbF_6_] (**1**)

Complex [(Mes)­Ru­(κ^3^ *N,N*′,*N″*-**L1**)]­[SbF_6_] (**1**) (**L1 =** *N*,*N*′-bis­(2,6-diisopropylphenyl)-*N″*-(2-pyridinylmethyl)­guanidinate) was prepared by treating the dimer [{(Mes)­RuCl}_2_(μ-Cl)_2_][Bibr ref17] with stoichiometric amounts of the ligand **HL1** and KOH in excess in methanol, in the presence of NaSbF_6_ (Scheme 2). By analogy with related systems,^14e^ the stepwise preparation of complex **1** via the chlorido complex [(Μes)­RuCl­(κ^2^ *N*,*N*′-**HL1**)]­[SbF_6_] (**2**) (Experimental Section, Figure S2, SI for X-ray data of complex **2**) was also attempted, but it resulted unsuccessful because the basicity of **HL1** prevented the formation of pure samples of **2**. A mixture of **2**, **1,** and the guanidinium compound **H**_**2**_ **L1­[SbF**_**6**_] in a molar ratio 40/30/30 was obtained (see SI page S6).

**Scheme 2:**
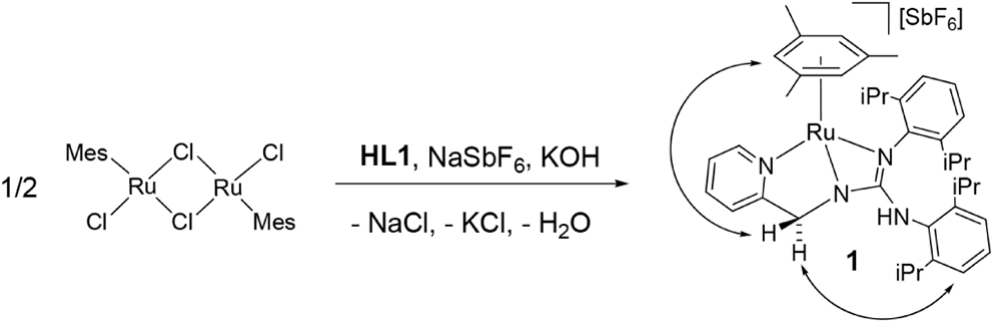
Preparation and Selected NOE of Complex **1**

Complex **1** was characterized by analytical and spectroscopic methods. An IR band at 3390 cm^–1^ and a ^1^H NMR singlet at 5.24 are assigned to the remaining NH functionality. Coordination of the pyridine is supported by the marked downfield shift of H_6_ (9.20 ppm in **1**; 8.41 ppm in **HL1**). Consistent with a stereogenic Ru center, the CH_2_ protons are diastereotopic, and their signals are observed at 3.99 and 3.35 ppm. NOESY correlations indicate that one CH_2_ proton is closer to the mesitylene ligand, whereas the other is oriented toward a 2,6-diisopropylphenyl ring and shielded by the aromatic ring current (Scheme 2).

In the crystal structure of **1** (Figure 1), the cation [(Mes)­Ru­(κ^3^ *N,N*′,*N*″-**L1**)]^+^ exhibits a semisandwich structure with an η^6^ coordination of mesitylene (Ru–CT 1.685(15) Å). Despite the small bite angles N19–Ru1–N17 [62.5(3)°] and N17–Ru1–N10 [74.9(3)°], **L1** features a κ^3^ *N,N*′,*N″* facial coordination (N10–Ru1 2.115(9); N17–Ru1 2.105(9); N19–Ru1 2.098(9) Å). Formation of two fused metallacycles enforces pyramidalization of N17 [Σ°_N17_ = 323.6(1.2)°], reminiscent of that previously observed in related phosphano-guanidine metal FLP complexes.^14a,14b,14e,14f^

**Figure 1:**
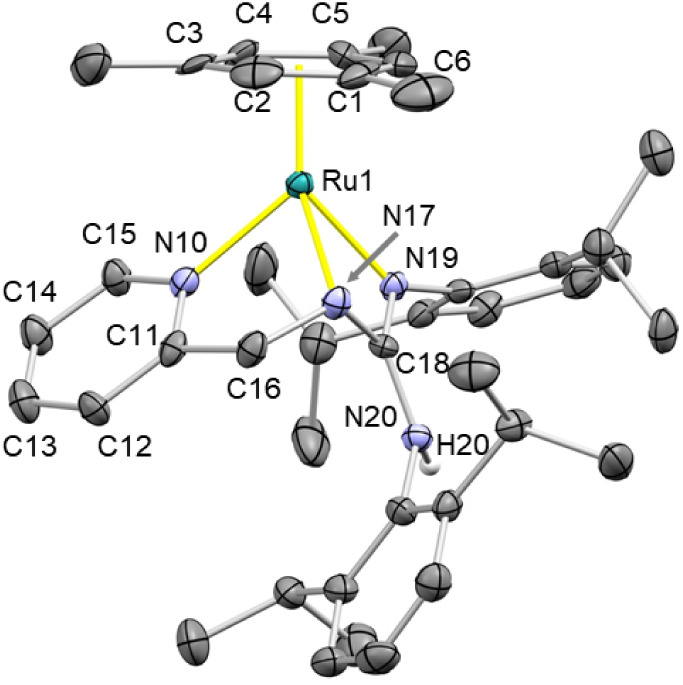
ORTEP view of the cation [(Mes)­Ru­(κ^3^ *N,N*′,*N*″-**L1**)]^+^ in **1**. Thermal ellipsoids are at 50% probability. Most hydrogen atoms are omitted for clarity. Selected bond lengths (Å) and angles (°) are Ru1–CT 1.685(15), N10–Ru1 2.115(9), N17–Ru1 2.105(9), N19–Ru1 2.098(9), N20–H20 0.899(10), C18–N17 1.360(13), C18–N19 1.311(13), C18–N20 1.366(13), N19–C18–N17 109.5(9), N19–C18–N20 123.8(9), N17–C18–N20 126.7(9), C18–N17–C16 119.0(9), C18–N17–Ru1 92.4(6), C16–N17–Ru1 112.2(6), N19–Ru1–N17 62.5(3), N19–Ru1–N10 89.4(3), N17–Ru1–N10 74.9(3), CT–Ru–N10 130.50(5), CT–Ru–N17 137.56(5), CT–Ru–N19 135.24­(5). CT, centroid of C1, C2, C3, C4, C5, and C6.

### Synthesis and Reactivity of the Complex [(Mes)­Ru­(κ^3^ *N,N*′,*N″*-L2)]­[SbF_6_] (**4**)

Reaction of the dimer [{(Mes)­RuCl}_2_(μ-Cl)_2_][Bibr ref17] with *N*-2-pyridinyl-*N*-(2-pyridinylmethyl)­amine (**HL2**) in the presence of NaSbF_6_ afforded the chlorido complex [(Mes)­RuCl­(κ^2^ *N*,*N*′-**HL2**)]­[SbF_6_] (**3**). Treatment of **3** with NaHCO_3_ yielded [(Mes)­Ru­(κ^3^ *N,N*′,*N″*-**L2**)]­[SbF_6_] (**4**), containing the tridentate amidate derived from **HL2** (Scheme 3). Lower basicity of the **HL2** ligand compared to **HL1** allows us to synthesize compound **4** via the chloride species **3**, containing the neutral **HL2** ligand.

**Scheme 3:**
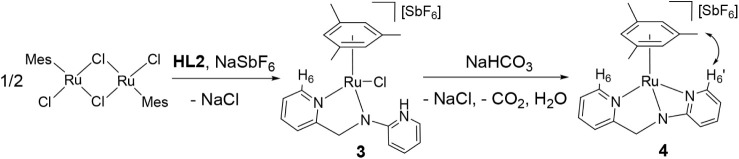
Synthetic Route and Selected NOE for Complex **4**

Complexes **3** and **4** were characterized by analytical and spectroscopic methods. Coordination of the pyridine nitrogen attached to the methylene group is indicated by downfield shifts of H_6_ (8.79 in **3**, 9.02 ppm in **4**; 8.55 in **HL2**). Complex **3** exhibits an IR band at 3264 cm^–1^ and a broad ^1^H resonance at 10.74 ppm, consistent with an NH functionality of the pyridinium group. Formation of **3** involves a [1,3]-proton shift from the amine nitrogen to the pyridinyl group. In contrast, complex **4** lacks NH signals and shows indicative NOE correlations between H_6′_ (but not with H_4′_) of the *N*-bound pyridine and mesitylene methyl protons (Scheme 3). These spectroscopic data for **4** are consistent with a four-membered Ru–N–C–N­(Py) metallacycle featuring a deprotonated *sp* ^3^ -N atom. The κ^2^ *N,N*′ (**3**) and facial κ^3^ *N,N*′,*N″* (**4**) coordination modes render a stereogenic ruthenium center, leading to diastereotopic methylene protons in both complexes (see Experimental Section). As for **3**, in agreement with the proposed structure, DFT calculations have been performed showing that structure **3** is 7.9 kcal·mol^–1^ more stable than the related amine tautomer **3′** (Figure 2).

**Figure 2:**
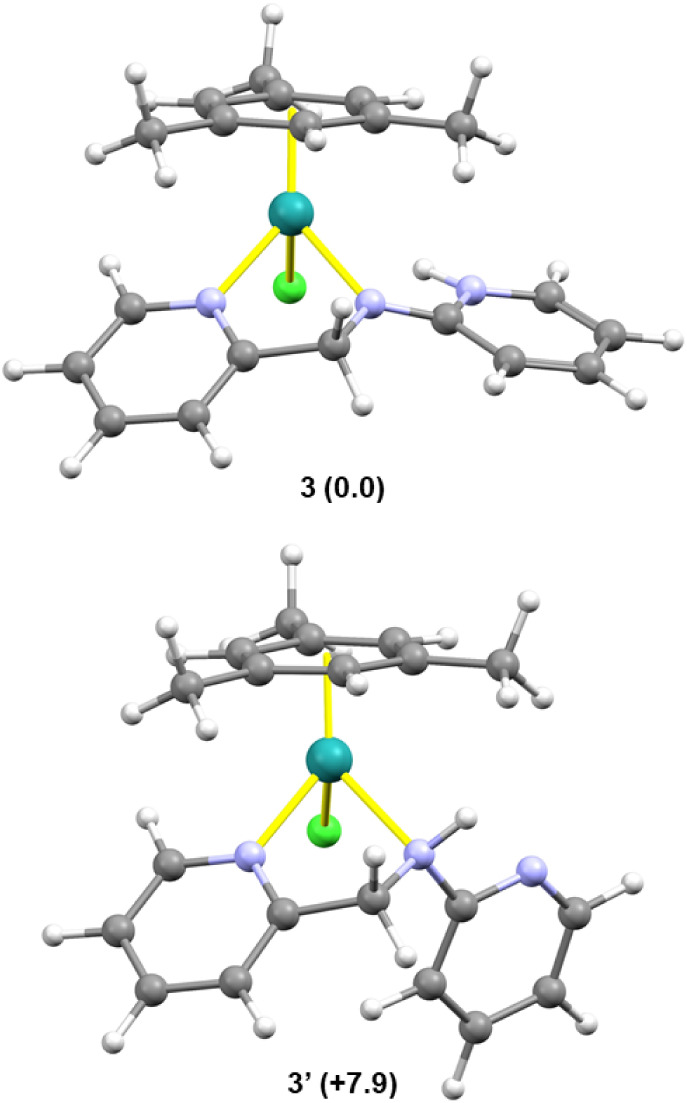
Calculated structures of **3** and its tautomer **3′** along with relative Gibbs free energy values (kcal·mol^–1^, B97D3/def2svp//B97D3/def2tzvp, CPCM/CH_2_Cl_2_, 298 K, 1 atm). Gray, carbon; white, hydrogen; green, chlorine; blue lavender, nitrogen; teal green, ruthenium.

On the other hand, the analysis of the bond lengths of the calculated structure **3,** along with the calculated bond order values (Figure 3), suggests that the resonance structure **II** should mostly contribute to the electronic structure of **3**.^18^ Also, the preliminary crystal structure of **3**, determined by single-crystal X-ray diffraction analysis, confirming the structure proposed for the cation [(Mes)­RuCl­(κ^2^ *N*,*N*′-**HL2**)]^+^ (see S3–S5, SI).

**Figure 3:**
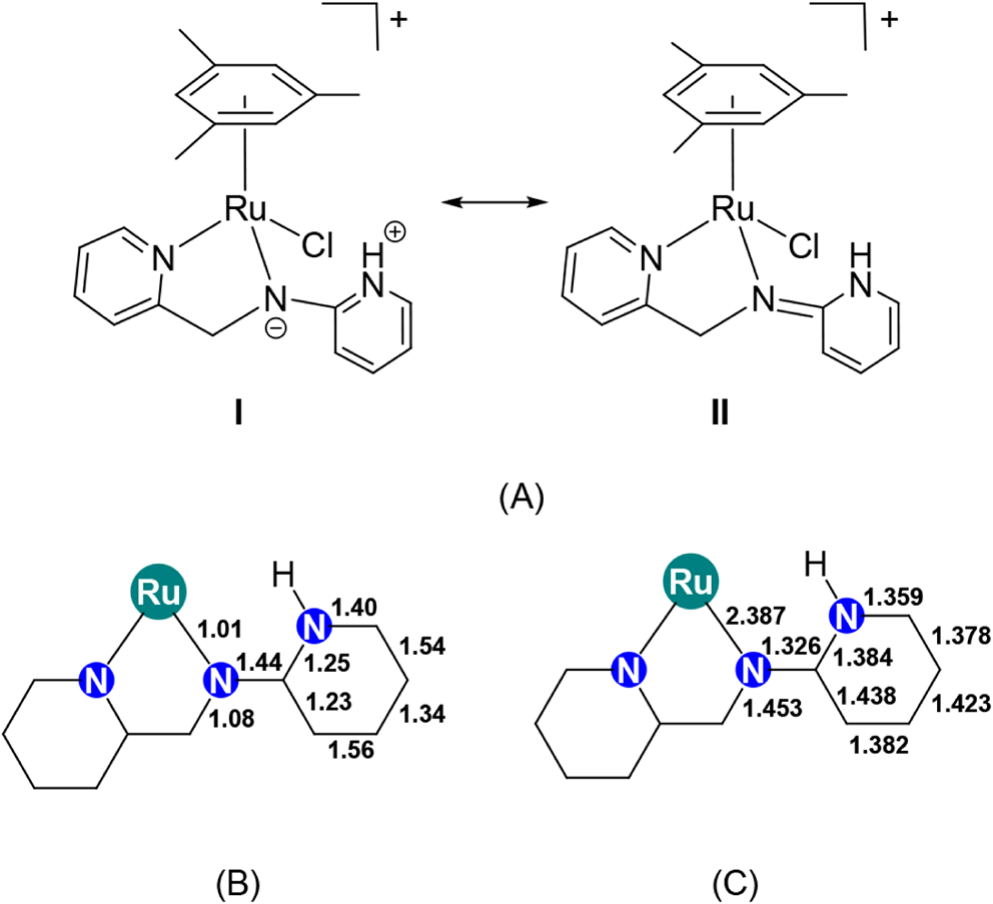
Resonance structures of **3** (A), along with a selection of calculated bond order values (B), selected bond lengths (Å) in the calculated structure of **3** (C).

To probe the stability of the Ru–N–C–N­(Py) metallacycle, protonation of **4** was examined. Treatment of **4** with HSbF_6_ in MeOH affords the *sp* ^3^-N-protonated complex **5** in 91% yield. Complex **5** can also be accessed from **3** via chloride abstraction with concomitant 1,3-prototropic tautomerization (Scheme 4).

**Scheme 4:**
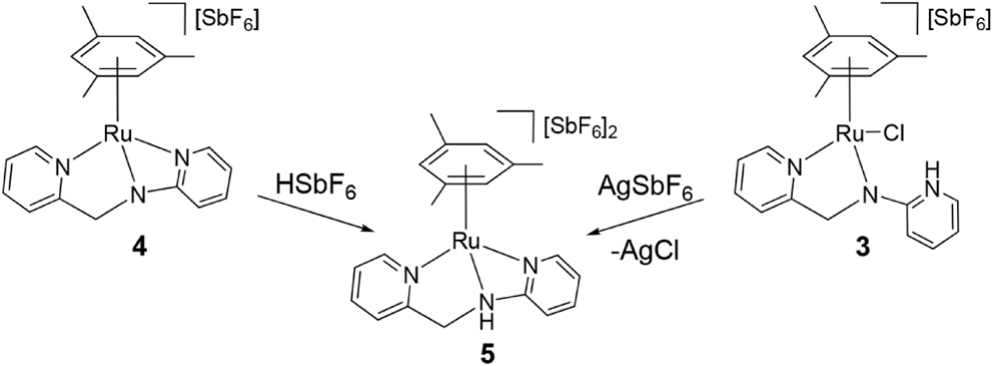
Formation of Complex **5**

Complex **5** was characterized by IR and NMR spectroscopy, elemental analysis, and X-ray crystallography (Figure 4). In the ^1^H NMR spectrum, the methylene group resonates as an AB system (δ = 5.17, 5.07 ppm, *J* = 16.8 Hz), additionally exhibiting coupling of both hydrogen atoms with the NH proton at 8.32 ppm (*J* = 3.6 Hz). In the crystal structure of **5** (Figure 4), [(Mes)­Ru­(κ^3^ *N,N*′,*N*″-**HL2**)]^2+^ contains an η^6^ mesitylene ligand (Ru–CT 1.689(9) Å) and a facial κ^3^ *N,N*′,*N*″ **HL2** ligand (N1–Ru 2.093(6), N10–Ru 2.100(6), N7a–Ru 2.124(11) Å) rendering an overall semisandwich structure. The – CH_2_–NH– fragment exhibits positional disorder (see S6, SI).

**Figure 4:**
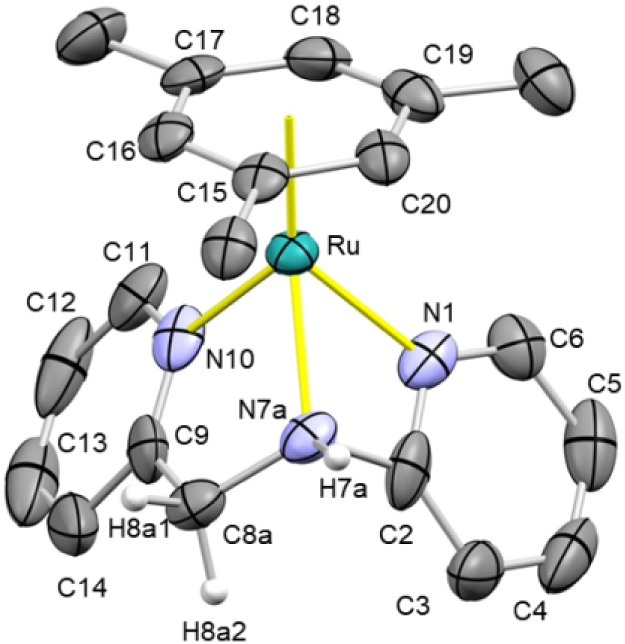
ORTEP view of the cation [(Mes)­Ru­(κ^3^ *N,N*′,*N*″-**HL2**)]^2+^ in **5**. Thermal ellipsoids are at 50% probability. Most hydrogen atoms are omitted for clarity. Selected bond lengths (Å) and angles (°) are Ru–CT 1.689(9), N1–Ru 2.093(6), N10–Ru 2.100(6), N7a–Ru 2.124(11), N1–Ru–N10 83.0(2), N1–Ru–N7a 61.0(3), N10–Ru–N7a 78.6(3), N1–Ru–CT 135.48(5), N7a–Ru–CT 136.26(5), N10–Ru–CT 134.21(5). CT, centroid of C15, C16, C17, C18, C19, and C20.

### Reactivity of Complexes **1** and **4** toward H_2_

Complexes **1** and **4** share a strained four-membered metallacycle. Cleavage of a Ru–N bond could generate a TMFLP featuring ruthenium as the Lewis acidic site and nitrogen as the Lewis basic site, enabling heterolytic splitting of H_2_. Accordingly, their reactivity toward H_2_ was investigated.

Exposure of THF-*d*_8_ solutions of **4** to 5 bar H_2_ at 20 °C for 39 h yields hydrido complexes of formula [(Mes)­RuH­(κ^2^ *N*,*N*′-**HL2**)]­[SbF_6_] (**7/7′**) in a **4**/**7/7′** molar ratio of 51/45/4.^19^ The composition remains unchanged for an additional 24 h. Hydride resonances appear at −5.48 (**7**) and −4.87 ppm (**7′**). NOESY data for **7** show correlations between the hydride signal and the N­(Py)H resonance (9.41 ppm) as well as H_6′_ (7.51 ppm) of the pyridinium moiety (Scheme 5). COSY confirms coupling between N­(Py)H and H_6′_. For minor **7′**, coupling between the methylene resonance, centered at 5.03 ppm, and an amine N­(CH_2_)H signal at 7.98 ppm supports the amino-like structure proposed for **7′** (Scheme 5).

**Scheme 5:**
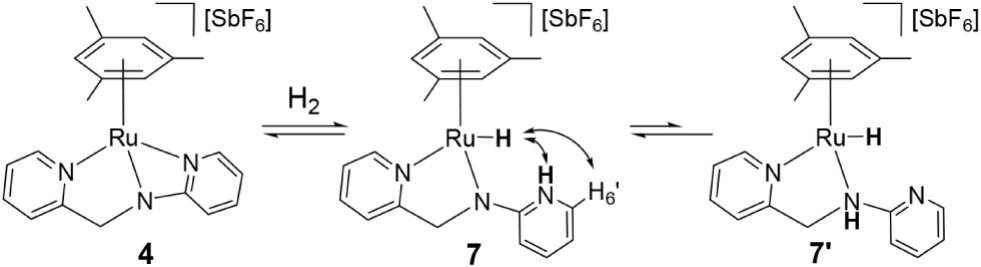
Reaction of Complex **4** with H_2_ and Selected NOE for **7**

In contrast, reaction of **1** with H_2_ (5 atm) over −50 to 90 °C does not yield detectable amounts of [(Mes)­RuH­(κ^2^ *N*,*N*′-**HL1**)]­[SbF_6_] (**6**), although **6** is observed in small amounts (**1**/**6** ≈ 98/2) under catalytic hydrogenation and dehydrogenation conditions (*vide infra*). Most probably, the higher basicity of the guanidinate ligand **L1** compared to the amidate ligand **L2**, and consequently the stronger ruthenium–nitrogen bond, is the reason why the hydride compound **6** is not observed. A high-field^1^H NMR signal at −5.36 ppm is assigned to the Ru–H moiety. The formulation of complex **6** (Scheme 6) is proposed by analogy with the related compound [(*p*-cymene)­RuH­(**HL**)]­[SbF_6_] (**HL** = *N*,*N*′-bis­(*p*-Tolyl)-*N*″-(2-pyridinylmethyl)­guanidine).^14g^

**Scheme 6:**
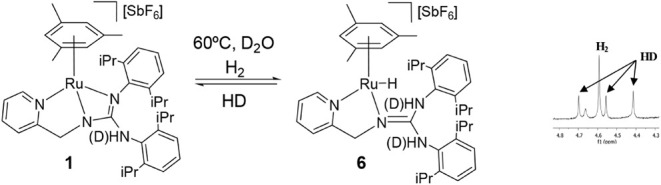
Reaction of Complex **1** with H_2_ in the Presence of D_2_O, and a Selected Region of the ^1^H NMR Spectrum Where the Presence of HD Is Observed

The hydrogen activation is reversible under mild conditions. Upon removal of H_2_, complex **4** is regenerated from a **4**/**7**/**7′** (51/45/4) mixture after *ca*. 12 h at room temperature. H/D exchange experiments in H_2_ further support the reversible H_2_ activation. The exposure of **1** (Scheme 6) or **4** (SI) to 5 atm H_2_ in THF-d_8_/D_2_O (0.45 mL/25 μL) at 60 °C (**1**) or room temperature (**4**) results in the formation of HD, consistent with the reversible H_2_ activation via hydride formation (Experimental Section). The formation of the HD indicates that, when compound **1** reacts with hydrogen, the hydride compound **6** is formed, even though this is not observed by NMR.

Notably, during the reaction of **1** with H_2_ in the presence of D_2_O, besides the formation of HD, H/D exchange at C_4_ and C_5_ of the pyridinyl group is observed (after 7 days: 64% and 8%, respectively). Accordingly, the reaction of **1** with D_2_ in THF-d_8_ at 90 °C resulted in extensive deuteration at both positions, C_4_ (87%) and C_5_ (86%), within 20 h (Scheme 7). Since no deuteration of the methylene group is detected, a pyridine dearomatization/rearomatization pathway is expected to be disfavored.^14g,20^ The observed deuteration pattern is consistent with equilibria involving **1-d** and intermediates **6′**/**6″**, where H/D is 1,5- or 1,4-transferred from the metal center to C_4_ or C_5_ of the pyridine moiety. An analogous mechanism was proposed by Gunanathan et al. for ruthenium-catalyzed pyridine hydroboration.^21^ Steric interactions between the pyridino C_6_ and an isopropyl substituent may suppress the more common 1,3-hydrogen transfer (Scheme 7).

**Scheme 7:**
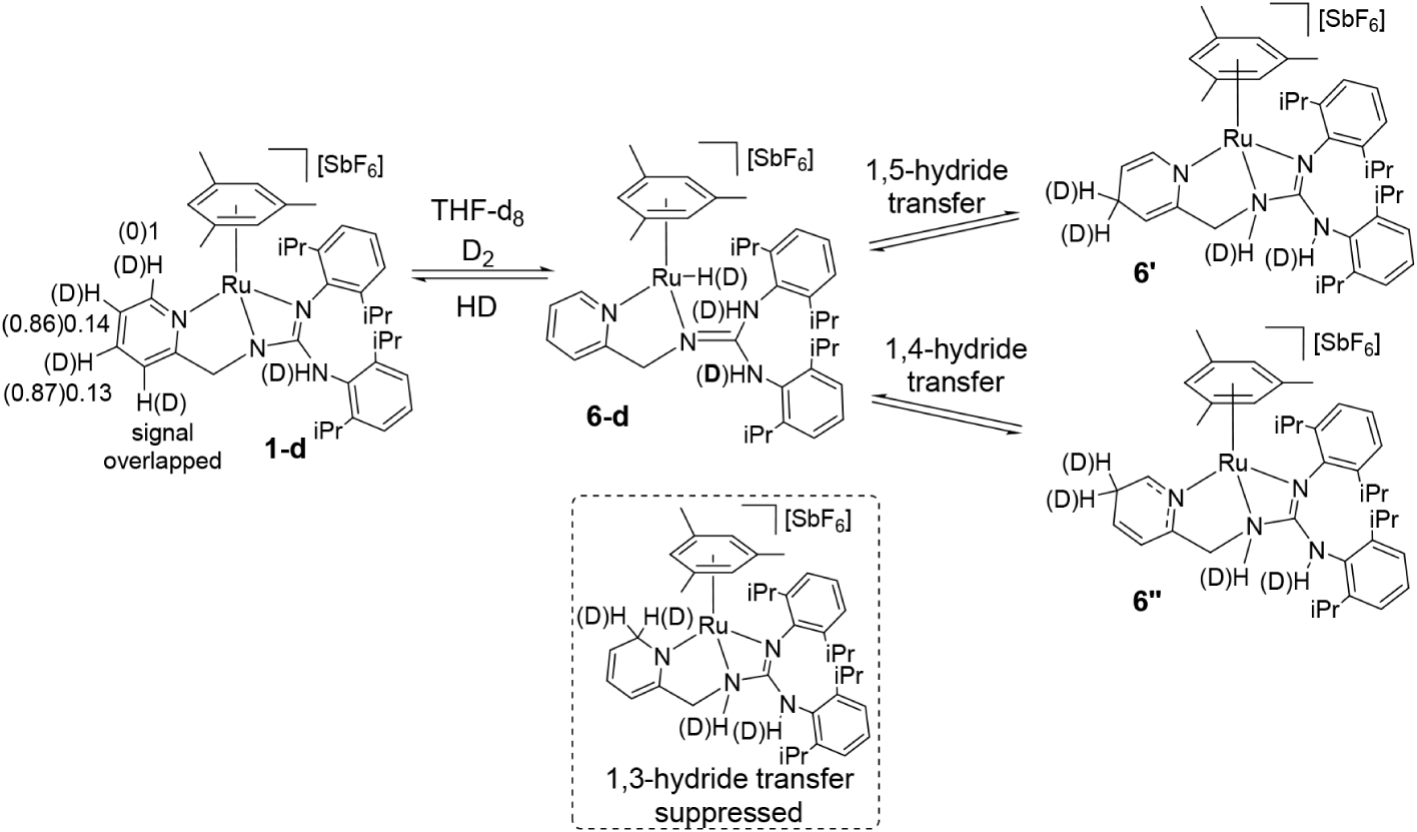
Reaction of Complex **1** with D_2_

For complex **4**, the observed rate of hydride formation (**7**/**7′**) is comparable to those reported for main group FLPs^22^ and TMFLPs.^23,14g^

### Hydrodechlorination Reactions

In the course of studies of hydrogenation of **1** using crystalline samples of **1**, formation of small amounts of the chlorido complex [(η^6^-Mes)­RuCl­(κ^2^ *N*,*N*′-**HL1**)]­[SbF_6_] (**2**) was detected. Since **1** crystallizes with a molecule of CH_2_Cl_2_ in the unit cell, we envisioned that **2** might form from the hydrido complex **6** via a hydrodechlorination of CH_2_Cl_2_ (Scheme 8a), by analogy with previously reported zirconium-based TMFLP systems^7a^ and iridium hydrido complexes^24^ [Cp*Ir­(bpy-OMe)­H]^+^ and [Cp*Ir­(bpy)­H]^+^.

**Scheme 8:**
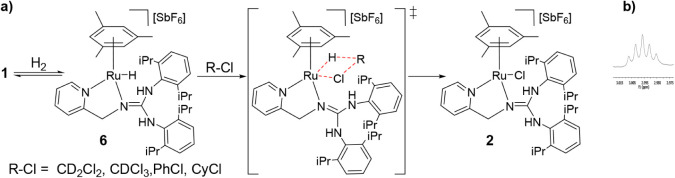
a) Proposed Pathway for the Dehydrochlorination Reaction Carried Out by **1;** b) ^1^H NMR Signal of CHD_2_Cl

Given the relevance of hydrodechlorination in synthetic organic chemistry and in the degradation of chlorinated organic compounds,^25^ this result prompted us to examine the ability of these systems to mediate hydrodechlorination reactions. Accordingly, the reactivity of **1** and **4** toward a range of chlorinated substrates, namely CD_2_Cl_2_, CDCl_3_, chlorobenzene, and cyclohexyl chloride, was investigated under H_2_. Complex **1** promotes hydrodechlorination under H_2_ at 90 °C (or 60 °C for CDCl_3_) over several hours (Table 1). Scheme 8a outlines a plausible reaction sequence for a generic chlorinated organic substrate R–Cl, whereas Scheme 8b shows the^1^H NMR signal of CHD_2_Cl corresponding to the hydrodechlorination product of CD_2_Cl_2_. Quantitative conversion of **1** into **2** (Table 1, entries 1, 3, and 4) supports the formation of hydride **6** from **1** and H_2_ under the reaction conditions. Similarly, complex **4** also promotes the hydrodechlorination of CD_2_Cl_2_ at room temperature (Table 1, entry 2). With respect to substrate structure, cleavage of the C–Cl bond is faster for the aromatic substrate chlorobenzene than for aliphatic chlorides (Table 1, entry 4), in agreement with previously reported studies.^25^

**Table 1:**
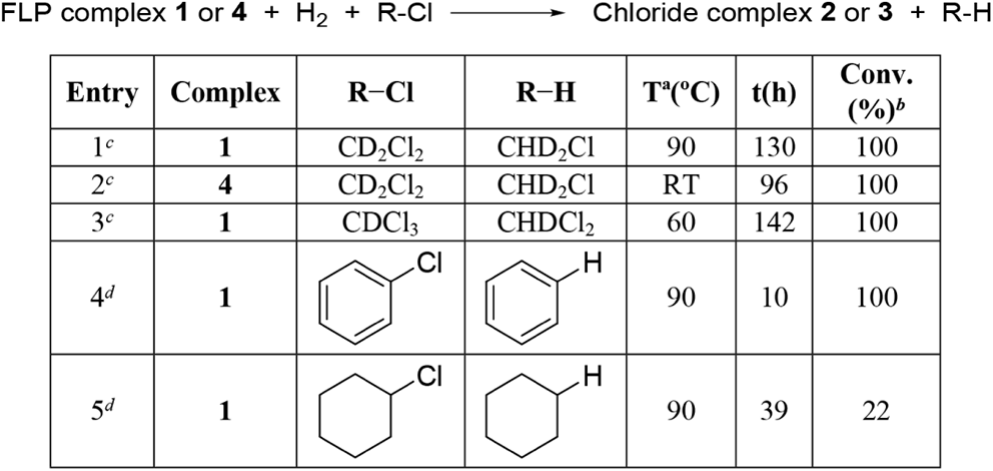
Hydrodechlorination Reaction^a^

a Reaction conditions: complex **1** or **4** (0.015 mmol) and H_2_ (5 atm).

b Based on complexes **2** or **3**. Determined by ^1^H NMR spectroscopy.

c 0.45 mL of chlorinated solvent.

d Substrate R–Cl (0.300 mmol) in 0.45 mL of THF-*d*_8_.

### Formic Acid Dehydrogenation Catalysis

In addition to the reversible formation of hydride species from H_2_, we investigated whether the complexes **1** and **4** could be engaged in dehydrogenation processes, using formic acid as a model substrate for base-free H_2_ release (Table 2). Complexes **1** and **4** catalyze the dehydrogenation of formic acid at 80 °C in THF-*d*_8_, in the absence of added base. At a catalyst/substrate molar ratio of 1/100, conversions of *ca*. 90% were obtained after 4 h for complex **1** and 23 h for complex **4** (Table 2, entries 1 and 2). For complex **1**, the presence of water in the reaction medium (THF-*d*_8_/D_2_O, 0.35/0.10 mL) enhances activity, giving 57% conversion after 1 h compared to 48% under anhydrous conditions (Table 2, entries 1 and 3). At higher conversions, or with increased water content (THF-*d*_8_/D_2_O, 0.10/0.35 mL), partial catalyst deactivation is observed (Table 2, entries 1, 3, and 4). In line with this behavior, ^1^H NMR spectra of aqueous reaction mixtures show the formation of the dicationic species [(η^6^-Mes)­Ru­(κ^2^ *N*,*N*′-**H**_**2**_ **L1** ^**+**^)]^2+^ (**H**_**2**_ **L1** ^**+**^ = *N*,*N*′-bis­(2,6-diisopropylphenyl)-*N″*-(2-pyridinylmethyl)­guanidinium), consistent with protonation of FLP complex **1** by formic acid under the reaction conditions.

**Table 2:**
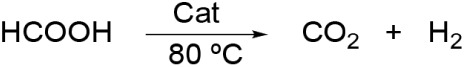
Formic Acid Dehydrogenation Catalysis^a^

Entry	Catalyst (mol %)	Solvent	*t* (h)	Conv. (%)^b^
1	1 (1)	THF-*d*_8_	1	48
			4	94
2	4 (1)	THF-*d*_8_	23	90
3	1 (1)	THF-*d*_8_/D_2_O (0.35 mL/0.1 mL)	1	57
			4	89
4	1 (1)	THF-*d*_8_/D_2_O (0.1 mL/0.35 mL)	10	89
5	1 (0.1)	THF-*d*_8_	1	26
			13	92

a Reaction conditions: HCOOH (0.30 mmol), internal reference 1,3,5-trimethoxybenzene, in 0.45 mL of THF-*d*_8_.

b Determined by ^1^H NMR spectroscopy based on 1,3,5-trimethoxybenzene.

The highest activity was observed for complex **1** at a substrate/catalyst molar ratio of 1000/1, affording 26% conversion within 1 h (TOF_1h_ = 260) (Table 2, entry 5). Although the overall activity is relatively modest, it is noteworthy that the transformation proceeds under base-free conditions.^26^ To assess the involvement of hydride species, reactions were monitored by ^1^H NMR spectroscopy. No hydrido complexes were detected during reactions conducted at 80 °C, however, when the reaction was carried out at 60 °C, the hydrido complex [(Mes)­RuH­(κ^2^ *N*,*N*′**-HL1**)]­[SbF_6_] (**6**) was observed in approximately 2% abundance for catalyst **1**, consistent with the formation of hydride intermediates under dehydrogenation conditions.

### Catalytic Hydrogenation Reactions

Having established that complexes **1** and **4** are competent in reversible H_2_ activation and dehydrogenation processes, we finally evaluated their performance in catalytic hydrogenation of selected unsaturated organic compounds. The hydrogenation of unsaturated organic compounds, including olefins, ketones, and imines, is of central importance in the petrochemical, agrochemical, and pharmaceutical industries.^27^ Over the past two decades, FLP-based catalysis has emerged as an alternative approach, initially demonstrated for C—N bond reductions^28^ and later extended to other substrates with notable findings for hydrogenation of C—C,^29,30^ C—O,^29,31^ and C—N^29,32^ bonds. Reports of catalytic hydrogenation mediated by TMFLPs remain scarce. Zirconium-based FLPs were shown to catalyze imine hydrogenation,^8b^ and Peters and coworkers reported efficient olefin hydrogenation with [^Mes^DPB^Ph^]­Ni.^33^ The use of complexes **1** and **4** represents a TMFLP strategy, in which a transition-metal center cooperates with a nitrogen donor to promote substrate activation.

As shown so far, the steric constraint prevents complete Ru–N quenching in complexes **1** and **4**, which might enable cooperative H_2_ activation^34^ (Scheme 9) and subsequent hydride transfer.^7a,35^ On this basis, complexes **1** and **4** were evaluated as catalysts for the hydrogenation of relevant functional groups in benchmark substrates, including the C—C bonds of acrylates,^27c,36^ the C—O bond of 2,2,2-trifluoroacetophenone,^27b,37,38,39^ and the C—N bond of *N*-benzylideneaniline.^27b,38a,40^ Reactions were typically carried out at 90 °C in THF-*d*_8_, using a catalyst/substrate molar ratio of 1/20 under 5 bar H_2_. Table 3 summarizes the selected results with conversions determined by ^1^H NMR spectroscopy. Quantitative conversions and, in most cases, conversions of around 90% were achieved within a few hours.

**Scheme 9:**
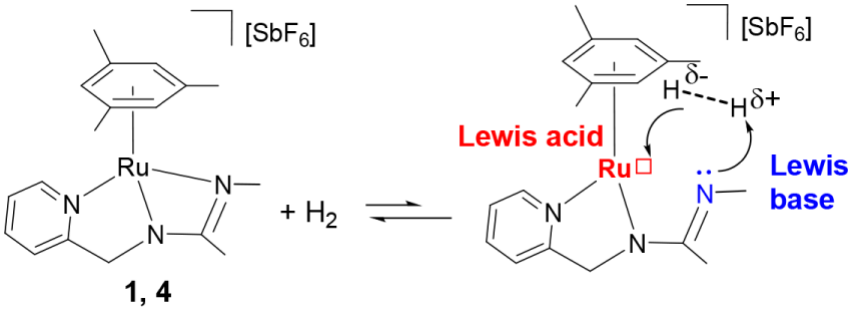
H_2_ Activation by Complexes **1** and **4**

**Table 3:**
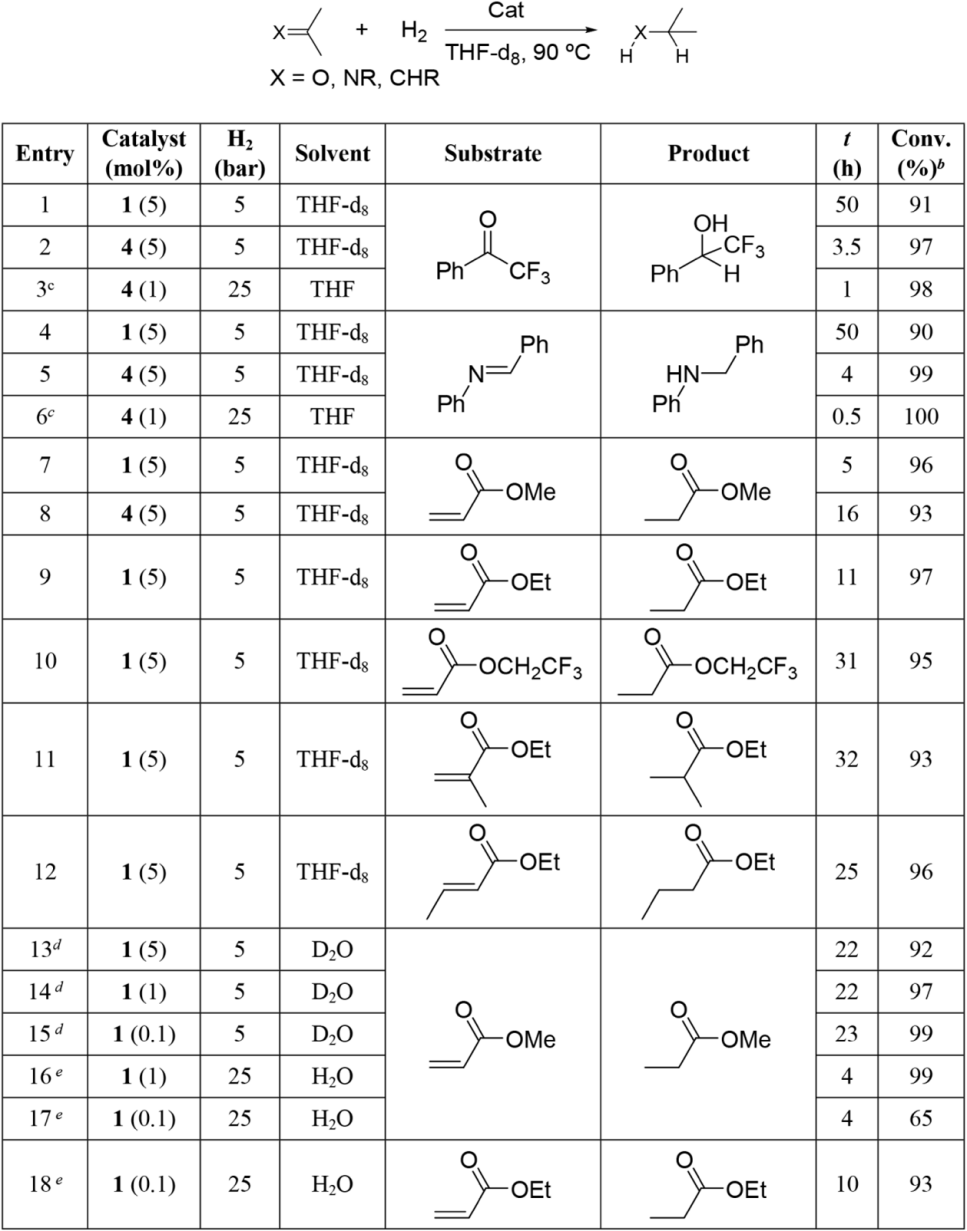
Hydrogenation of Unsaturated Substrates^a^

a Reaction conditions: catalyst 0.015 mmol (5 mol %), substrate (0.30 mmol), H_2_ (5 bar) in 0.45 mL of THF-*d*_8_.

b Determined by ^1^H NMR spectroscopy using 1,3,5-trimethoxybenzene (0.03 mol) as internal reference.

c Catalyst 0.03 mmol (1 mol %), substrate (3.0 mmol), in 4.5 mL of THF.

d Reaction conditions: catalyst 0.015–0.0003 mmol (5–0.1 mol %), substrate (0.30 mmol), H_2_ (5 bar), in 0.45 mL of D_2_O.

e Catalyst 0.03–0.003 mmol (1–0.1 mol %), substrate (3.0 mmol), H_2_ (25 bar), internal reference 1,3,5-trimethoxybenzene (0.3 mmol), in 4.5 mL of H_2_O.

Complex **1** showed higher activity than **4** for methyl acrylate hydrogenation, affording conversions above 90% after 5 and 16 h, respectively (Table 3, entries 7 and 8). However, catalyst **4** is most active in the reactions of 2,2,2-trifluoroacetophenone and *N*-benzylideneaniline, reaching conversions close to 100% in 3.5 and 4 h, respectively (Table 3, entries 1 and 2 and entries 4 and 5). Under higher hydrogen pressure (25 bar) and with a catalyst/substrate ratio of 1/100, full conversion is achieved within 1 (C—O) and 0.5 h (C—N) (Table 3, entries 3 and 6). Although these activities are lower than those reported for state-of-the-art homogeneous ruthenium hydrogenation catalysts,^27c,36,37,38,39,40^ they compare favorably with related TMFLP systems, for substituted imines reported by Wass and coworkers,^8b^ and with representative main-group FLPs applied to similar substrates (methyl acrylate,^29,30^ acetophenone,^29,31^ and *N*-benzylideneaniline^29,32^ ).

The scope of acrylate hydrogenation with complex **1** was further explored. Increased steric bulk at the ester group reduces the reaction rate, likely due to steric interactions with the iPr substituents of the ligand (Table 3, entries 7, 9, and 10). Substitution at the α- and β-positions of the acrylate similarly slows hydrogenation (Table 3, entries 11 and 12). It is noteworthy that, although methyl acrylate is prone to polymerization during hydrogenation at relatively high temperatures, no acrylate polymerization was observed during the reactions.^36^

Given the environmental impact of organic solvents, the hydrogenation of acrylates was also examined in water as a solvent using catalyst **1**. Under 5 bar of H_2_, catalyst/substrate molar ratios of 1/20, 1/100, and 1/1000 afforded conversions above 90% after 22 h (Table 3, entries 13–15). The apparently similar activity observed at lower catalyst loadings is consistent with the limited solubility of **1** in water. Increasing the hydrogen pressure to 25 bar further enhances activity, leading to quantitative conversion within 4 h (Table 3, entries 14 and 16). For ethyl acrylate, effective hydrogenation in water is maintained at catalyst loadings as low as 0.1 mol % under 25 bar of H_2_ (Table 3, entries 9 and 18). Notably, whereas related pyridinyl-guanidinato ruthenium complex [(*p*-cymene)­Ru­(**HL**)]­[SbF_6_] (**HL** = *N*,*N*′-bis­(*p*-Tolyl)-*N*″-(2-pyridinylmethyl)­guanidinate) are prone to side reactions that compromise catalytic performance,^14g^ complexes **1** and **4** show no detectable degradation under the hydrogenation conditions employed.

Finally, it should be noted that during the course of the catalytic reactions in THF-*d*_8_, catalysts **1** and **4** are observed almost exclusively. Only in the hydrogenation reaction of *N*-benzylideneaniline catalyzed by **1** (Table 3, entry 4), and in the hydrogenation of methyl acrylate catalyzed by **4** (Table 3, entry 8) are the corresponding hydride complexes **6** and **7** detected, and only in minor amounts (ca. 2%). This observation suggests that hydride formation constitutes the rate-determining step under the reaction conditions. Consistent with this interpretation, increasing the hydrogen pressure from 5 to 25 bar enhances reaction rates in all cases examined (Table 3, entries 2 and 4; 5 and 6; 14 and 16).

## Conclusion

In summary, pyridinyl-guanidine **HL1** and pyridinyl-amine **HL2** ligands provide a suitable platform for the construction of ruthenium-based frustrated Lewis pairs, in which the metal center acts as the Lewis acidic component and the nitrogen donor as the basic counterpart. The resulting complexes **1** and **4** are capable of reversible H_2_ activation, giving access to hydride species (**6** and **7**/**7′**) that are consistent with their subsequent reactivity. In the presence of H_2_, these TMFLP systems engage in hydride-transfer processes, as exemplified by the hydrodechlorination of chlorinated substrates. In addition, both complexes exhibit catalytic activity in the base-free dehydrogenation of formic acid, highlighting the reversibility of hydrogen activation and the accessibility of hydrogen-transfer equilibria within this framework.

Finally, complexes **1** and **4** are stable TMFLP catalysts for hydrogenation reactions. In particular, complex **1** efficiently catalyzes the hydrogenation of C—C bonds in acrylates, including reactions conducted in water as solvent, while both complexes are effective in the hydrogenation of C—O and C—N bonds in representative ketone and imine substrates. Taken together, these results underscore the versatility of this ruthenium–nitrogen TMFLP platform in mediating a broad range of hydrogen-transfer reactions.

## Experimental Section

No uncommon hazards are noted during the research work. All preparations have been carried out under argon. All solvents were treated in a PS-400-6 Innovative Technologies Solvent Purification System (SPS) and degassed before use. Infrared spectra were recorded on a PerkinElmer Spectrum-100 (ATR mode) FT-IR spectrometer. Carbon, hydrogen, and nitrogen analyses were performed using a PerkinElmer 240 B microanalyser. ^1^H and ^13^C spectra were recorded on a Bruker AV-300 spectrometer (300.13 MHz), Bruker AV-400 (400.16 MHz) or Bruker AV-500 (500.13 MHz). In both ^1^H NMR and ^13^C NMR measurements, the chemical shifts are expressed in ppm downfield from SiMe_4_. *J* values are given in Hz. NOESY, ^1^H, and ^13^C correlation spectra were obtained using standard procedures. Mass spectra were obtained with a Micro Tof-Q Bruker Daltonics spectrometer. The high-pressure reactions were carried out in a laboratory Parallel synthesizer autoclave polymerization reactor (316SS + PTFE), 30 mL × 6, 120 °C, 90 bar.

### Preparation of the Guanidine Ligand **HL1**


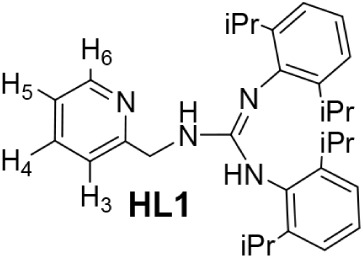


At RT, a mixture of 2-aminoethyl-pyridine (510.0 μL, 5.00 mmol) and bis­(2,6-diisopropylphenyl)-carbodiimide (1849.4 mg, 5.00 mmol) in dry THF (10 mL) was stirred for 20 h. The resulting solution was vacuum-evaporated to dryness. The residue was washed with *n*-hexane (3 × 5 mL). Evaporation of the solvent under vacuum gave the pyridinyl-guanidine compound as a white solid. Crystals of **HL1** suitable for X-ray diffraction analysis were obtained by crystallization from CH_2_Cl_2_/*n*-hexane solutions. Yield: 2201.3 mg, 94%. Anal. Calcd for C_31_H_42_N_4_: C, 79.11; H, 8.99; N, 11.90. Found: C, 79.16; H, 9.03; N, 12.15. HRMS (μ-TOF), C_31_H_43_N_4_, [M + H]^+^, calcd: 471.3482, found: 471.3489. IR (cm^–1^): ν­(NH) 3436, 3376 (w), ν­(N—C) 1629 (m). ^1^H NMR (500.10 MHz, CD_2_Cl_2_, RT): δ = 8.41 (bs, 1H, H_6_ (Py)), 7.64 (bt, *J* = 7.2 Hz, 1H, H_4_ (Py)), 7.41 (bd, *J* = 7.5 Hz, 1H, H_3_ (Py)), 7.30 (m, 1H, Ar), 7.19, 7.12 (2 × d, *J* = 7.4 Hz, 4H, Ar), 7.13 (overlapped, 1H, H_5_ (Py)), 6.97 (bt, *J* = 7.3 Hz, 1H, Ar), 4.92 (s, 1H, N*H*Ar), 4.63 (s, 2H, CH_2_), 4.58 (s, 1H, N*H*CH_2_), 3.29 (m, 2H, CH (iPr)), 3.22 (m, 2H, CH (iPr)), 1.30, 1.10 (2 × d, *J* = 6.3 Hz, 12H, Me), 1.24, 1.06 (2 × bd, *J* = 4.9 Hz, 12H, Me). ^13^C­{^1^H} NMR (125.77 MHz, CD_2_Cl_2_, RT): δ = 160.13 (*C*CH_2_), 149.53 (CH_6_ (Py)), 148.95, 141.94, 145.21, 132.45 (*C*iPr), 147.35 (C—N), 136.85 (CH_4_ (Py)), 129.31, 124.69, 123.63, 123.05 (Ar), 122.64 (CH_3_ (Py)), 122.47 (CH_5_ (Py)), 47.22 (CH_2_), 28.98 (CH (iPr)), 25.08, 24.97, 24.03, 23.07 (Me).

### Preparation of the Pyridinyl-Amidine Ligand **HL2**


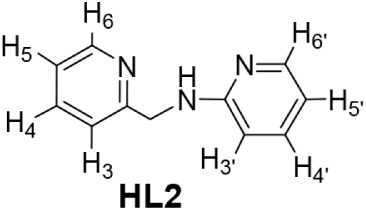


In a round-bottomed flask, at room temperature, 2-aminopyridine (5.00 g, 53.13 mmol), 2-pyridinecarboxaldehyde (5.05 mL, *d* = 1.126 g/mL, 53.13 mmol), and toluene (100 mL) were added. The round-bottomed flask was then equipped with a reflux condenser and a Dean–Stark, and the mixture was boiled under reflux for 96 h. The resulting solution was cooled at RT and then added to a mixture of NaBH_4_ (4.00 g, 105.73 mmol) and 20 mL of dry EtOH at 0 °C. The resulting suspension was stirred for 6 h, and then, 150 mL of dichloromethane was added. The solution was washed with a saturated NaHCO_3_ aqueous solution (6 × 20 mL). The organic solution was dried with MgSO_4_, filtered, and dried under a vacuum. The residue was extracted with ethanol (80 mL) and vacuum-dried. Then the solid obtained was extracted with *n*-heptane (150 mL), and the resulting solution was vacuum-dried, affording a white solid. Yield: 4.62 g, 47%. Anal. Calcd for C_11_H_11_N_3_: C, 71.30; H, 6.00; N, 22.70. Found: C, 71.00; H, 5.50; N, 22.60. HRMS (μ-TOF), C_11_H_12_N_3_ [M + H]^+^, calcd: 186.1026, found: 186.1026. IR (cm^–1^): ν­(NH) 3233 (m), ν­(N—C) 1599, 1585 (s). ^1^H NMR (500.10 MHz, (CD_2_Cl_2_, RT): δ = 8.55 (d, *J* = 5.0 Hz, 1H, H_6_(PyCH_2_)), 8.07 (d, 1H, *J* = 5.0 Hz, H_6_(PyNH)), 7.65 (td, *J* = 7.7 Hz, *J* = 1.8 Hz, 1H, H_4_(PyCH_2_)), 7.40 (td, *J* = 7.7 Hz, *J* = 1.9 Hz, 1H, H_4_(PyNH)), 7.33 (d, *J* = 7.8 Hz, 1H, H_3_(PyCH_2_)), 7.18 (t, *J* = 6.1 Hz, 1H, H_5_(PyCH_2_)), 6.57 (t, *J* = 8.5 Hz, 1H, H_5_(PyNH)), 6.48 (d, *J* = 8.0 Hz, 1H, H_3_(PyNH)), 5.64 (s, 1H, NH), 4.62 (d, 2H, CH_2_). ^13^C­{^1^H} NMR (125.77 MHz, (CD_3_)_2_CO, RT): δ = 159.42 (C_2_(PyCH_2_)), 159.23 (C_2_(PyNH)), 149.84 (C_6_(PyNH)), 148.89 (C_6_(PyCH_2_)), 137.89 (C_4_(PyCH_2_)), 137.23 (C_4_(PyNH)), 122.78 (C_5_(PyNH)), 122.36 (C_3_(PyNH)), 113.68 (C_5_(PyCH_2_)), 108.42 (C_3_(PyCH_2_)), 47.88 (CH_2_).

### Preparation of the Complex [(Mes)­Ru­(κ^3^ *N*,*N*′,*N″*-L1)]­[SbF_6_] (**1**)


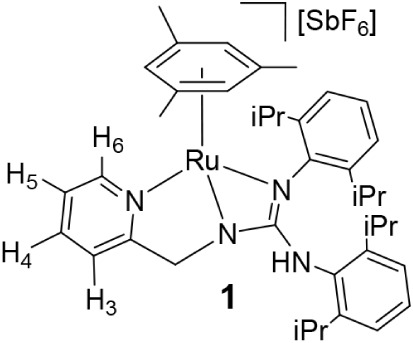


To a solution of the dimer [{(Mes)­RuCl}_2_(*μ-*Cl)_2_] (250.0 mg, 0.43 mmol) in methanol (20 mL), **HL1** (573.8, 0.86 mmol), NaSbF_6_ (222.5 mg, 0.86 mmol), and KOH in methanol (6.0 mL, 0.20 M, 1.20 mmol) were added. The resulting suspension was stirred for 24 h and then vacuum-evaporated to dryness. The residue was extracted with dichloromethane, and the resulting solution was concentrated under reduced pressure to *ca*. 2 mL. The slow addition of *n*-pentane led to the precipitation of an orange solid, which was washed with *n*-pentane (3 × 5 mL) and vacuum-dried. Crystals suitable for X-ray diffraction analysis were obtained by crystallization from CH_2_Cl_2_/methanol/*n*-pentane solutions. Yield: 766.9 mg, 95%. Calcd for C_40_H_53_N_4_RuSbF_6_: C, 51.85; H, 5.76; N, 6.04. Found: C, 51.74; H, 5.91; N, 6.08; HRMS (μ-TOF), C_40_H_53_N_4_Ru, [M – SbF_6_]^+^, calcd: 691.3319, found: 691.3306. IR (cm^–1^): ν­(NH) 3390 (w), ν­(N—C) 1559 (m), ν­(SbF_6_) 656 (s). ^1^H NMR (500.10 MHz, CD_2_Cl_2_, RT): δ = 9.20 (d, *J* = 6.2 Hz, 1H, H_6_(Py)), 7.82 (t, *J* = 4.3 Hz, 1H, H_4_(Py)), 7.54 (t, *J* = 6.6 Hz, 1H, H_5_(Py)), 7.35–7.05 (m, 6H, Ar), 6.97 (d, *J* = 7.8 Hz, 1H, H_3_(Py)), 5.24 (s, 1H, NH), 5.05 (s, 3H, C_6_ *H*_3_Me_3_), 3.99, 3.35 (AB system, *J* = 16.2 Hz, 2H, CH_2_), 3.42, 3.27, 2.49, 2.07 (4 × sept, 4H, CH­(iPr)), 1.95 (s, 9H, C_6_H_3_ *Me*_3_), 1.55, 1.50, 1.33, 1.18, 0.84, 0.75, 0.69 (8 × d, *J* = 6.8 Hz, 24H, Me). ^13^C­{^1^H} NMR (125.77 MHz, CD_2_Cl_2_, RT): δ = 167.35 (*C*CH_2_), 166.72 (C—N), 154.99 (CH_6_(Py)), 146.48, 146.38, 145.12, 143.52 (*C*iPr), 139.84 (CH_4_(Py)), 133.98, 130.85, 129.00, 127.94, 126.34, 125.67, 125.14, 124.41 (Ar), 124.95 (CH_5_(Py)), 122.06 (CH_3_(Py)), 102.44 (C­(C_6_H_3_Me_3_)), 78.22 (CH­(C_6_H_3_Me_3_)), 59.26 (CH_2_), 29.37, 28.85, 28.72, 28.44 (C (iPr)), 27.02, 26.88, 26.19, 24.93, 24.84, 24.71, 23.82, 22.88 (Me), 19.34 (C_6_H_3_ *Me*_3_).

### Preparation of the Complex [(Μes)­RuCl­(κ^2^ *N*,*N*′-HL1)]­[SbF_6_] (**2**)


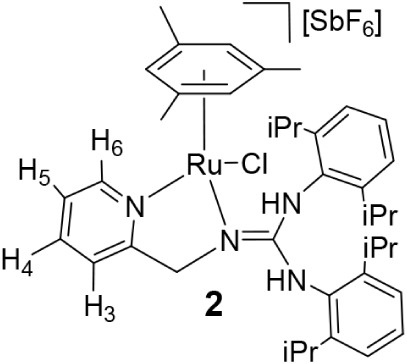


The dimer [{(Μes)­RuCl}_2_(μ-Cl)_2_] (250.0 mg, 0.43 mmol) and NaSbF_6_ (222.6 mg, 0.86 mmol) were added to a solution of the **HL1** ligand (402.8 mg, 0.86 mmol) in MeOH (10 mL). The orange solution was stirred under argon for 24 h, at 67 °C. The resulting suspension was vacuum-evaporated until dryness, and the residue was extracted with CH_2_Cl_2_ (20 mL). The solution was vacuum-evaporated until ca. 2 mL, and *n*-pentane (10 mL) was added, resulting in the precipitation of an orange solid. The suspension was decanted to remove *n*-pentane, and the solid was washed with *n*-pentane (2 × 10 mL) and vacuum-dried. A mixture of [(Μes)­RuCl­(κ^2^ *N*,*N*′-**HL1**)]­[SbF_6_] (**2**, 88%), [(Mes)­Ru­(κ^3^ *N*,*N*′,*N″*-**L1**)]­[SbF_6_] (**1**, 6%), and [**H**_**2**_ **L1**]­[SbF_6_] (6%) was obtained. The complex **2** was recrystallized from CH_2_Cl_2_/Et_2_O solutions. Single crystals of the complex were obtained by slow diffusion of Et_2_O into a CH_2_Cl_2_ solution. Yield: 681.3 mg, 81%. Calcd for C_40_H_54_N_4_ClRuSbF_6_: C, 49.88; H, 5.65; N, 5.82. Found: C, 50.10; H, 5.90; N, 5.90. HRMS (μ-TOF), C_40_H_54_N_4_RuCl, [M – SbF_6_]^+^, calcd: 727.3080, found: 727.3090. IR (cm^–1^): ν­(NH) 3348 (w), ν­(N—C) 1610 (m), ν­(SbF_6_) 654 (s). ^1^H NMR (500.10 MHz, CD_2_Cl_2_, RT): δ = 8.68 (d, *J* = 5.2 Hz, 1H, H_6_(Py)), 7.74 (t, *J* = 7.9 Hz, 1H, H_4_(Py)), 7.59 (s, 1H, NH), 7.32 (t, *J* = 7.6 Hz, 1H, H_5_(Py)), 7.43 (m, 3H, Ar), 7.33 (m, 3H, Ar), 7.17 (d, *J* = 7.0 Hz, 2H, Ar), 6.70 (d, 1H, H_3_(Py)), 5.79 (s, 1H, NH), 5.14 (s, 3H, C_6_ *H*_3_Me_3_), 4.37, 4.17 (AB system, *J* = 16.6 Hz, 2H, CH_2_), 3.58, 3.49, 3.25, 2.66 (4 × sept, 4H, CH­(iPr)), 2.21 (s, 9H, C_6_H_3_ *Me*_3_), 1.51, 1.50, 1.39, 1.25, 1.17, 1.15, 0.95, 0.62 (8 × d, *J* = 7.0 Hz, 24H, Me). ^13^C­{^1^H} NMR (125.77 MHz, CD_2_Cl_2_, RT): δ = 162.00 (*C*CH_2_), 155.12 (C—N), 154.20 (CH_6_(Py)), 149.51, 148.74, 147.05, 146.94 (*C*iPr), 139.66 (CH_4_(Py)), 133.02, 130.86, 130.76, 130.65, 125.99, 125.57, 125.29, 125.07 (Ar), 125.82 (CH_5_(Py)), 120.84 (CH_3_(Py)), 106.75 (C­(C_6_H_3_Me_3_)), 78.13 (CH­(C_6_H_3_Me_3_)), 60.41 (CH_2_), 29.81, 29.37, 29.18, 28.97 (CH­(iPr)), 28.72, 28.03, 26.46, 24.13, 24.01, 23.73, 22.50 (Me), 19.50 (C_6_H_3_ *Me*_3_).

### Preparation of the Complex [(Mes)­RuCl­(κ^2^ *N*,*N*′-HL2)]­[SbF_6_] (**3**)


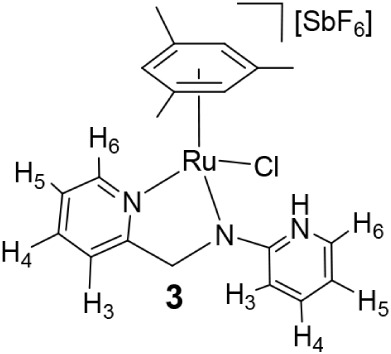


To a suspension of the dimer [{(Mes)­RuCl}_2_(*μ-*Cl)_2_] (1500.0 mg, 2.57 mmol), in methanol (20 mL), 950.9 mg (5.13 mmol) of **HL2** and 1328.2 mg (5.13 mmol) of NaSbF_6_ were added. The resulting solution was stirred for 2 days and vacuum-evaporated to dryness. The residue was extracted with dichloromethane, and the solution was concentrated under reduced pressure to ca. 2 mL. The slow addition of *n*-hexane led to the precipitation of an orange solid, which was washed with *n*-hexane (3 × 10 mL) and vacuum-dried. Crystals of **3** suitable for X-ray diffraction analysis were obtained by crystallization from CH_2_Cl_2_/*n*-hexane solutions. Yield: 2801.2 mg, 81%. Anal. Calcd for C_22_H_23_N_3_ClF_6_RuSb: C, 35.4; H, 3.4; N, 6.2. Found: C, 35.3; H, 3.2; N, 6.4. HRMS (μ-TOF), C_22_H_22_N_3_Ru, [M–SbF_6_–Cl–H], calcd: 406.0857, found: 406.0859. IR (cm^–1^): ν­(NH) 3264 (w), ν­(N = C) 1641, 1598 (m), ν­(SbF_6_) 649 (s). ^1^H NMR (500.10 MHz, CD_2_Cl_2_, RT): δ = 10.74 (s, 1H, NH), 8.79 (bd, 1H, H_6_ (PyCH_2_)), 7.89 (td, *J* = 7.8 Hz, *J* = 1.6 Hz, 1H, H_4_ (PyCH_2_)), 7.48 (t, *J* = 6.4 Hz, 1H, H_5_ (PyCH_2_)), 7.45 (d, 1H, H_3_ (PyCH_2_)), 7.40 (m, 1H, H_4_ (PyNH)), 7.36 (t, 1H, *J* = 6.3 Hz, H_6_ (PyNH)), 6.98 (bd, *J* = 9.4 Hz, 1H, H_3_ (PyNH)), 6.29 (tt, *J* = 6.6 Hz, *J* = 1.2 Hz, 1H, H_5_ (PyNH)), 5.11 (s, 3H, C_6_ *H*_3_Me_3_), 4.91, 4.85 (AB system, *J* = 16.8 Hz, 2H, CH_2_), 2.04 (s, 9H, Me). ^13^C­{^1^H} NMR (125.77 MHz, CD_2_Cl_2_, RT): δ = 162.63 (C_2_ (PyCH_2_)), 158.75 (C_2_ (PyNH)), 154.38 (C_6_ (PyCH_2_)), 140.53 (C_4_ (PyNH)), 139.87 (C_4_ (PyCH_2_)), 134.91 (C_6_(PyNH)), 126.06 (C_5_ (PyCH_2_)), 121.55 (C_3_ (PyCH_2_)), 115.54 (C_3_(PyNH)), 107.77 (C_5_ (PyNH)), 107.67 (C (C_6_H_3_Me_3_)), 77.78 (CH (C_6_H_3_Me_3_)), 62.04 (CH_2_), 19.49 (Me).

### Preparation of the Complex [(Mes)­Ru­(κ^3^ *N*,*N*′,*N″*-L2)]­[SbF_6_] (**4**)


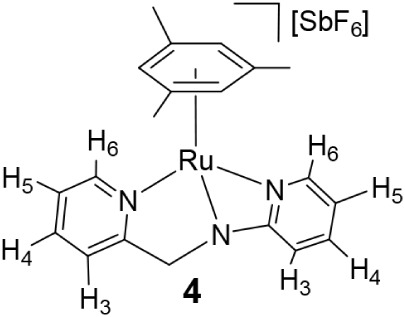


To a solution of the complex [(Mes)­RuCl­(κ^2^ *N*,*N*′-**HL2**)]­[SbF_6_] (**3**) (677.7 mg, 1.48 mmol) in methanol (20 mL), 124.0 mg (1.48 mmol) of solid NaHCO_3_ were added. The resulting suspension was stirred for 24 h and then vacuum-evaporated to dryness. The residue was extracted with dichloromethane and the resulting solution was concentrated under pressure to *ca*. 2 mL. The slow addition of *n*-hexane led to the precipitation of a brown solid, which was washed with *n*-hexane (3 × 10 mL) and vacuum-dried. Yield: 850.3 mg, 90%. Anal. Calcd for C_20_H_22_N_3_F_6_RuSb: C, 37.5; H, 3.5; N, 6.6. Found: C, 37.3; H, 3.5; N, 6.6. HRMS (μ-TOF), C_20_H_22_N_3_Ru, [M–SbF_6_], calcd: 406.0857, found: 406.0861. IR (cm^–1^): ν­(N—C) 1592 (m), ν­(SbF_6_) 652 (s). ^1^H NMR (500.10 MHz, CD_2_Cl_2_, RT): δ = 9.02 (d, *J* = 5.5 Hz, 1H, H_6_ (PyCH_2_)), 8.15 (d, 1H, *J* = 5.7 Hz, H_6_ (PyN)), 7.74 (td, *J* = 7.7 Hz, *J* = 1.5 Hz, 1H, H_4_ (PyCH_2_)), 7.37 (t, *J* = 7.9 Hz, 1H, H_4_ (PyN)), 7.34 (d, *J* = 7.7 Hz, 1H, H_3_ (PyCH_2_)), 7.29 (t, *J* = 6.6 Hz, 1H, H_5_ (PyCH_2_)), 6.61 (t, *J* = 6.2 Hz, 1H, H_5_ (PyN)), 6.47 (d, *J* = 8.5 Hz, 1H, H_3_ (PyN)), 5.15 (s, 3H, C_6_ *H*_3_Me_3_), 4.57, 4.50 (AB system, *J* = 17.6 Hz, 2H, CH_2_), 2.27 (s, 9H, Me). ^13^C­{^1^H} NMR (125.77 MHz, CD_2_Cl_2_, RT): δ = 179.81 (C_2_ (PyN)), 167.73 (C_2_ (PyCH_2_)), 154.86 (C_6_ (PyCH_2_)), 147.07 (C_6_ (PyN)), 139.62 (C_4_ (PyCH_2_)), 138.68 (C_4_ (PyN)), 124.83 (C_5_ (PyCH_2_)), 122.66 (C_3_ (PyCH_2_)), 115.82 (C_5_ (PyN)), 113.76 (C_3_ (PyN)), 104.56 (C (C_6_H_3_Me_3_)), 77.79 (CH (C_6_H_3_Me_3_)), 64.27 (CH_2_), 19.91 (Me).

### Preparation of the Complex [(Mes)­Ru­(κ^3^ *N*,*N*′,*N″*-HL2)]­[SbF_6_]_**2**_ **(5)**


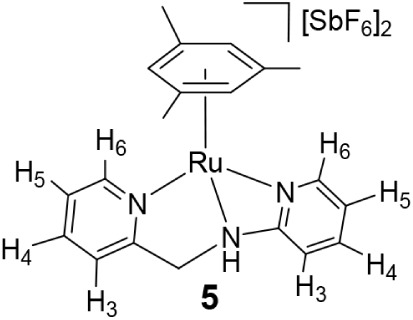


To a solution of the complex [(Mes)­Ru­(κ^3^ *N*,*N*′,*N″*-**L2**)]­[SbF_6_] (**4**) (400.0 mg, 0.62 mmol), in methanol (15 mL), HSbF_6 •_ 6 H_2_O (75 μL, 0.62 mmol) was added. The resulting solution was stirred for 6 h and concentrated under reduced pressure to ca. 2 mL. The slow addition of *n*-pentane led to the precipitation of a yellow solid, which was washed with *n*-pentane (3 × 10 mL) and vacuum-dried. Complex **5** was prepared by an alternative route by treating the chlorido complex **3** (50.0 mg, 0.07 mmol) with AgSbF_6_ (25.4 mg, 0.07 mmol) in acetone. The resulting suspension was filtered, and the filtrate was vacuum-evaporated until ca. 1 mL. The addition of *n*-pentane (10 mL) led to the precipitation of a yellow solid. Yellow crystals of **5** suitable for X-ray diffraction analysis were obtained by crystallization from acetone/*n*-pentane. Yield: 144 mg, 91%. Anal. Calcd for C_20_H_23_N_3_F_12_RuSb_2_: C, 27.4; H, 2.6; N, 4.8. Found: C, 27.4; H, 2.8; N, 4.7. HRMS (μ-TOF), C_20_H_22_N_3_Ru, [M–2SbF_6_–H], calcd: 406.0857, found: 406.0873. IR (cm^–1^): ν­(NH) 3262 (w), ν­(N = C) 1611 (m), ν­(SbF_6_) 653 (s). ^1^H NMR (500.10 MHz, (CD_3_)_2_CO, RT): δ = 9.58 (br d, 1H, *J* = 5.5 Hz, H_6_ (PyNH)), 8.87 (bd, *J* = 5.2 Hz, 1H, H_6_ (PyCH_2_)), 8.32 (s, 1H, NH), 8.23 (td, *J* = 7.9 Hz, *J* = 1.4 Hz, 1H, H_4_ (PyCH_2_)), 8.15 (td, *J* = 7.8 Hz, *J* = 1.4 Hz, 1H, H_4_ (PyNH)), 7.79 (d, 1H, H_3_ (PyCH_2_)), 7.74 (t, *J* = 5.8 Hz, 1H, H_5_ (PyCH_2_)), 7.73 (t, *J* = 5.9 Hz, 1H, H_5_ (PyNH)), 7.66 (d, 1H, H_3_ (PyNH)), 5.97 (s, 3H, C_6_ *H*_3_Me_3_), 5.17 (dd, *J* = 16.8 Hz, *J* = 3.6 Hz, 1H, C*H*H), 5.07 (d, *J* = 16.8 Hz, 1H, CH*H*), 2.55 (s, 9H, Me). ^13^C­{^1^H} NMR (125.77 MHz, (CD_3_)_2_CO, RT): δ = 163.72 (C_2_ (PyCH_2_)), 160.00 (C_2_ (PyNH)), 156.15 (C_6_ (PyNH)), 150.45 (C_6_ (PyCH_2_)), 144.42 (C_4_ (PyCH_2_)), 142.53 (C_4_ (PyNH)), 129.23 (C_5_ (PyCH_2_)), 127.87 (C_5_ (PyNH)), 125.97 (C_3_ (PyCH_2_)), 123.01 (C_3_ (PyNH)), 109.90 (C (C_6_H_3_Me_3_)), 79.13 (CH (C_6_H_3_Me_3_)), 61.07 (CH_2_), 20.20 (Me).

### Reaction of the Complexes [(Mes)­Ru­(κ^3^ *N*,*N*′,*N″*-L)]­[SbF_6_] (**L** = **L1** (**1**), **L2** (**4**)) with Hydrogen


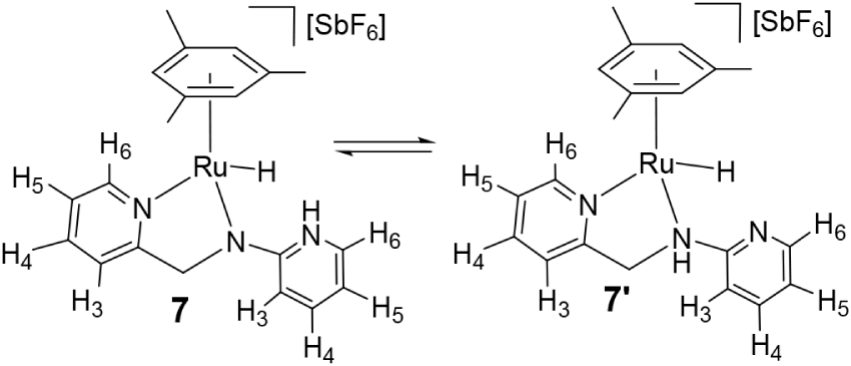


A high-pressure NMR tube containing a solution of the corresponding complex [(Mes)­Ru­(κ^3^ *N*,*N*′,*N″*-**L**)]­[SbF_6_] (**L** = **L1** (**1**), **L2** (**4**)) (0.015 mmol) in THF-*d*_8_ (0.45 mL), was pressurized with H_2_ (5 bar) and the resulting solution was monitored by NMR spectroscopy. After 39 h at RT (complex **4**), a conversion of 49% (complex [(Mes)­RuH­(κ^2^ *N*,*N*′-**HL2**)]­[SbF_6_] (**7**, 45%; **7′**, 4%) was achieved.^19^ The resulting hydrido complexes were characterized by NMR spectroscopy, under H_2_ pressure. The corresponding hydrido complex [(Mes)­RuH­(κ^2^ *N*,*N*′-**HL1**)]­[SbF_6_] (**6**) is not observed in the temperature range of −50 to 90 °C. **Complex 7.** ^1^H NMR (500.10 MHz, THF-*d*_8_, RT): δ = 9.41 (bs, 1H, NH), 8.58 (d, 1H, *J* = 6.0 Hz, H_6_ (PyCH_2_)), 7.82 (t, *J* = 7.7 Hz, 1H, H_4_ (PyCH_2_)), 7.51 (d, *J* = 7.4 Hz, 1H, H_6_ (PyNH)), 7.47 (d, *J* = 8.1 Hz, 1H, H_3_ (PyCH_2_)), 7.28 (t, *J* = 6.7 Hz, 1H, H_5_ (PyCH_2_)), 7.25 (td, *J* = 8.1 Hz, 1H, H_4_ (PyNH)), 7.12 (d, *J* = 9.1 Hz, 1H, H_3_ (PyNH)), 6.11 (t, *J* = 6.6 Hz, 1H, H_5_ (PyNH)), 5.23 (s, 3H, C_6_ *H*_3_Me_3_), 5.23, 4.92 (AB system, *J* = 17.2 Hz, 2H, CH_2_), 2.06 (s, 9H, Me), −5.48 (s, 1H, Ru–H). ^13^C­{^1^H} NMR (125.77 MHz, THF-*d*_8_, RT): δ = 162.53 (C_2_ (PyCH_2_)), 157.82 (C_2_ (PyN)), 155.19 (C_6_ (PyCH_2_)), 138.54 (C_4_ (PyN)), 138.16 (C_4_ (PyCH_2_)), 135.08 (C_3_ (PyCH_2_)), 124.29 (C_5_ (PyCH_2_)), 120.69 (C_6_ (PyN)), 113.95 (C_3_ (PyN)), 105.61 (C_5_ (PyN)), 105.50 (C (C_6_H_3_Me_3_)), 79.54 (CH (C_6_H_3_Me_3_)), 61.48 (CH_2_), 19.63 (Me). **Complex 7′.** ^1^H NMR (500.10 MHz, THF-*d*_8_, RT): δ = 7.98 (bt, 1H, *J* = 8.2 Hz, NH), 5.38 (s, 3H, C_6_ *H*_3_Me_3_), 5.03 (m, 2H, CH_2_), 2.05 (s, 3H, Me), −4.87 (s, Ru–H). ^13^C­{^1^H} NMR (125.77 MHz, THF-*d*_8_, RT): δ = 107.34 (C (C_6_H_3_Me_3_)), 18.59 (Me).

### Reaction of the Complexes **1** and **4** with H_2_In the Presence of D_2_O

A high-pressure NMR tube containing a solution of the corresponding complex [(Mes)­Ru­(κ^3^ *N*,*N*′,*N″*-**L**)]­[SbF_6_] (**L** = **L1** (**1**), **L2** (**4**)) (0.015 mmol) in THF-*d*_8_/D_2_O: 0.45 mL/25 μL, was pressurized with H_2_ (5 bar) and the resulting solution was monitored by NMR spectroscopy. After 15 h at 60 °C (complex **1**) or 3 days at RT (complex **4**), HD was formed in the reaction medium.

### Reaction of the Complex **1** with D_2_

A high-pressure NMR tube containing a solution of complex [(Mes)­Ru­(κ^3^ *N*,*N*′,*N″*-**L1**)]­[SbF_6_] (**1**) (13.9 mg, 0.015 mmol) in THF-*d*_8_ (0.45 mL), was pressurized with D_2_ (5 bar). The resulting solution was heated at 90 °C and monitored by ^1^H NMR spectroscopy.

### Hydrodechlorination Reactions

In a high-pressure NMR tube containing the corresponding complex [(Mes)­Ru­(κ^3^ *N*,*N*′,*N″*-**L**)]­[SbF_6_] (**L** = **L1** (**1**), **L2** (**4**)) (0.015 mmol), the chlorinated organic compound was added (CD_2_Cl_2_, 0.45 mL; CDCl_3_, 0.45 mL; chlorobenzene, 30.5 μL (0.300 mmol) in THF-*d*_8_ (0.45 mL); cyclohexyl chloride 35.5 μL (0.300 mmol) in THF-*d*_8_ (0.45 mL)). The resulting solution was pressurized with H_2_ (5 bar), heated at 90 °C or at 60 °C (**1**) or at RT (**4**), and monitored by ^1^H NMR spectroscopy.

### Formic Acid Dehydrogenation


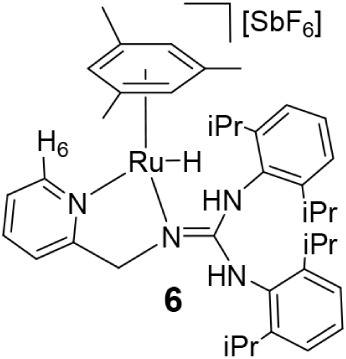


A high-pressure NMR tube containing a solution of catalyst (0.015–0.0003 mmol), HCOOH (11 μL, 0.300 mmol) and 1,3,5-trimethoxybenzene (5 mg, 0.030 mmol) in THF-*d*_8_/D_2_O (0.45 mL/0 mL–0.10 mL/0.35 mL) was heated at the appropriate temperature. To control the water content in the reaction medium, we have used THF-*d*_8_ dried over 4 Å MS and intentionally added defined amounts of D_2_O. The resulting solution was monitored by ^1^H NMR spectroscopy. The formation of hydrido complex [(Mes)­RuH­(κ^2^ *N*,*N*′-**HL1**)]­[SbF_6_] (**6**) is detected (**1**/**6** = 98/2) during the course catalytic dehydrogenation. **Complex 6**: ^1^H NMR (500.10 MHz, THF-*d*_8_, RT): δ = 8.57 (d, *J* = 5.6 Hz, 1H, H_6_ (Py)), 5.18 (s, 3H, C_6_ *H*_3_Me_3_), 2.19 (s, 9H, C_6_H_3_ *Me*_3_), 1.05, 0.54 (2 × d, *J* = 6.9 Hz, 6H, Me), −5.36 (*s*, 1H, Ru–H).

### General Procedure for the Catalytic Hydrogenation Reactions under 5 bar of H_2_

A high-pressure NMR tube containing the catalyst (0.015–0.0003 mmol) and the substrate to be hydrogenated (0.30 mmol) in THF-*d*_8_ or D_2_O (0.45 mL) was pressurized with hydrogen gas (5 bar). The tube was heated at the appropriate temperature, and the solution was monitored by ^1^H NMR. Conversion values were determined by ^1^H NMR.

### General Procedure for the Catalytic Hydrogenation Reactions under 25 bar of H_2_

A high-pressure reactor containing the catalyst (0.03–0.003 mmol) and the substrate to be hydrogenated (3.0 mmol) in THF or H_2_O (4.5 mL) was pressurized with hydrogen gas (25 bar). The tube was heated at the appropriate temperature. After cooling to room temperature, the vessel was vented and opened. Samples of 0.1 mL of THF solutions were evaporated, dissolved in 0.45 mL of THF-*d*_8_, and analyzed by ^1^H NMR. ^1^H NMR analyzed samples of 0.1 mL of water solutions in 0.35 mL of THF-d8. Conversion values were determined by ^1^H NMR.

### Crystal Structure Determination

X-ray diffraction data were collected at 100(2) K on an APEX-DUO (**2**, **5**, **HL1**) or D8 VENTURE (**1**) Bruker diffractometers with graphite-monochromated Mo Kα radiation (λ = 0.71073 Å) using ω- and φ-scans. Intensities were integrated and corrected for absorption effects with SAINT-PLUS^41^ and SADABS^42^ programs, both included in the APEX4 package. The structures were solved by the Patterson method with SHELXS-97 ^43^ and refined by full matrix least-squares on F^2^ with SHELXL-2014 ^44^ under WinGX.^45^

### Crystal Data and Structure Refinement for **1**

C_81_H_108_Cl_2_F_12_N_8_Ru_2_Sb_2_, 1938.29 g·mol^–1^, monoclinic, *P*2_1_/*c*, *a* = 14.2135(5) Å, *b* = 20.6536(7) Å, *c* = 28.6290(10) Å, β = 90.4430(10)°, *V* = 8404.1(5) Å^3^, *Z* = 4, *D*_calc_ = 1.532 g·cm^–3^, μ = 1.126 mm^–1^, *F*(000) = 3928, 0.230 × 0.180 × 0.120 mm^3^, θ_min_/θ_max_ 1.875/25.027°, index ranges −16 ≤ *h* ≤ 16, −24 ≤ *k* ≤ 24, −34 ≤ *l* ≤ 34, reflections collected/independent 214576/14822 [*R*(int) = 0.0383], *T*_max_/*T*_min_ 0.7458/0.6929, data/restraints/parameters 14822/16/995, Goof­(*F* ^2^) = 1.163, *R*_1_ = 0.1043 [*I* > 2·σ­(*I*)], w*R*_2_ = 0.2579 (all data), largest diff. peak/hole 8.455/–1.230 e·Å^–3^. CCDC deposit number 2395014.

### Crystal Data and Structure Refinement for **2**

C_40_H_54_ClF_6_N_4_RuSb, 963.14 g·mol^–1^, orthorhombic, *P*2_1_2_1_2_1_, *a* = 10.5513(9) Å, *b* = 15.7384(13) Å, *c* = 24.481(2) Å, *V* = 4065.3(6) Å^3^, *Z* = 4, *D*_calc_ = 1.574 g·cm^–3^, μ = 1.163 mm^–1^, *F*(000) = 1952, 0.250 × 0.240 × 0.180 mm^3^, θ_min_/θ_max_ 1.538/29.605°, index ranges −14 ≤ *h* ≤ 14, −21 ≤ *k* ≤ 20, −33 ≤ *l* ≤ 33, reflections collected/independent 43341/10886 [*R*(int) = 0.0326], *T*_max_/*T*_min_ 0.7459/0.6614, data/restraints/parameters 10886/21/531, Goof­(*F* ^2^) = 1.024, *R*_1_ = 0.0244 [*I* > 2·σ­(*I*)], *wR*_2_ = 0.0541 (all data), absolute structure parameter −0.019(6), largest diff. peak/hole 0.734/–0.310 e·Å^–3^. CCDC deposit number 2395012.

### Crystal Data and Structure Refinement for **5**

C_29_H_44_F_12_N_3_RuSb_2_, 1007.24 g·mol^–1^, monoclinic, *P*2_1_/*n*, *a* = 8.4815(10) Å, *b* = 19.040(2) Å, *c* = 22.675(3) Å, β = 98.657(2)°, *V* = 3620.0(7) Å^3^, *Z* = 4, *D*_calc_ = 1.848 g·cm^–3^, μ = 1.980 mm^–1^, *F*(000) = 1972, 0.240 × 0.060 × 0.050 mm^3^, θ_min_/θ_max_ 1.403/25.681°, index ranges −10 ≤ *h* ≤ 10, −23 ≤ *k* ≤ 23, −27 ≤ *l* ≤ 26, reflections collected/independent 30445/6883 [*R*(int) = 0.0542], *T*_max_/*T*_min_ 0.8131/0.6658, data/restraints/parameters 6883/2/362, Goof­(*F* ^2^) = 1.048, *R*_1_ = 0.0562 [I > 2·σ­(I)], w*R*_2_ = 0.1536 (all data), largest diff. peak/hole 3.984/–1.885 e·Å^–3^. CCDC deposit number 2395013.

### Crystal Data and Structure Refinement for **H_2_L1**

C_31_H_42_N_4_, 470.68 g·mol^–1^, triclinic, *P*1̅, *a* = 9.0637(6) Å, *b* = 16.6304(12) Å, *c* = 21.3159(15) Å, α= 67.2720(10)°, β = 89.3970(10)°, γ = 74.6240(10)°, *V* = 2842.1(3) Å^3^, *Z* = 4, *D*_calc_ = 1.100 g·cm^–3^, μ = 0.065 mm^–1^, *F*(000) = 1024, 0.350 × 0.250 × 0.200 mm^3^, θ_min_/θ_max_ 1.041/26.372°, index ranges −11 ≤ *h* ≤ 11, −20 ≤ *k* ≤ 20, −26 ≤ *l* ≤ 25, reflections collected/independent 25998/11632 [*R*(int) = 0.0214], *T*_max_/*T*_min_ 0.9804/0.9161, data/restraints/parameters 11632/0/695, Goof­(*F* ^2^) = 1.015, *R*_1_ = 0.0401 [*I* > 2·σ­(*I*)], *wR*_2_ = 0.1019 (all data), largest diff. peak/hole 0.488/–0.249 e·Å^–3^. CCDC deposit number 2395010.

### DFT Calculations

Molecular structure optimizations and frequency calculations were carried out with Gaussian16 (revision C.01)^46^ using the method B97D3,^47^ including the D3 dispersion correction scheme by Grimme with Becke–Johnson damping.^48^ The def2svp^49^ basis and pseudo potential were used for all atoms, and the “ultrafine” grid was employed in all calculations. Stationary points were characterized by vibrational analysis. The structures were optimized in dichloromethane (298 K, 1 atm) using the CPCM method.^50^ Finally, energy values were refined at the level B97D3/def2tzvp^49^//B97D3/def2svp. Bond order values were calculated at the level B97D3/def2tzvp using Multiwfn.^51^

## Supplementary Material



## Notes

NMR spectra of compounds **HL1**, **HL2**, **1–7**, crystallographic data of ligand **HL1** and complexes **2**, **3**, and **5**; and coordinates of calculated structures (PDF)
