# Supplementary material for: Ruthenium Complexes Containing Pyridinyl-Derived Ligands as FLP Catalysts
Source: Inorg Chem. 2026 May 14;65(20):11180–93. doi: 10.1021/acs.inorgchem.6c00833 (PMC13213901; doi:10.1021/acs.inorgchem.6c00833)
Supplement: Supplementary file 1 [file ic6c00833_si_001.pdf]

Ruthenium Complexes containing Pyridinyl-derived Ligands as FLP Catalysts

Alejandro Grasa, Hannah Middlebrook, Réka Anna Józsa, Vincenzo Passarelli,* Fernando Viguri, Ricardo Rodríguez* and Pilar Lamata*

Instituto de Síntesis Química y Catálisis Homogénea (ISQCH), CSIC - Universidad de Zaragoza, Departamento de Química Inorgánica, Pedro Cerbuna 12, 50009 Zaragoza, Spain

Corresponding Authors

E-mail addresses:

plamata@unizar.es, riromar@unizar.es, passarel@unizar.es

Table of Contents **page**

1 NMR spectra of HL1, HL2 and 1-7 **S2**

2 X-ray crystallography **S11**

3 File .res of 3 **S15**

4 Coordinates of calculated structures of 3 and 3' **S25**

5 References **S28**

1 NMR spectra of HL1, HL2 and 1-7 and Scheme S1

* Denotes solvent

^{1}H NMR of HL1 (500.10 MHz, CD_2Cl_2, RT)

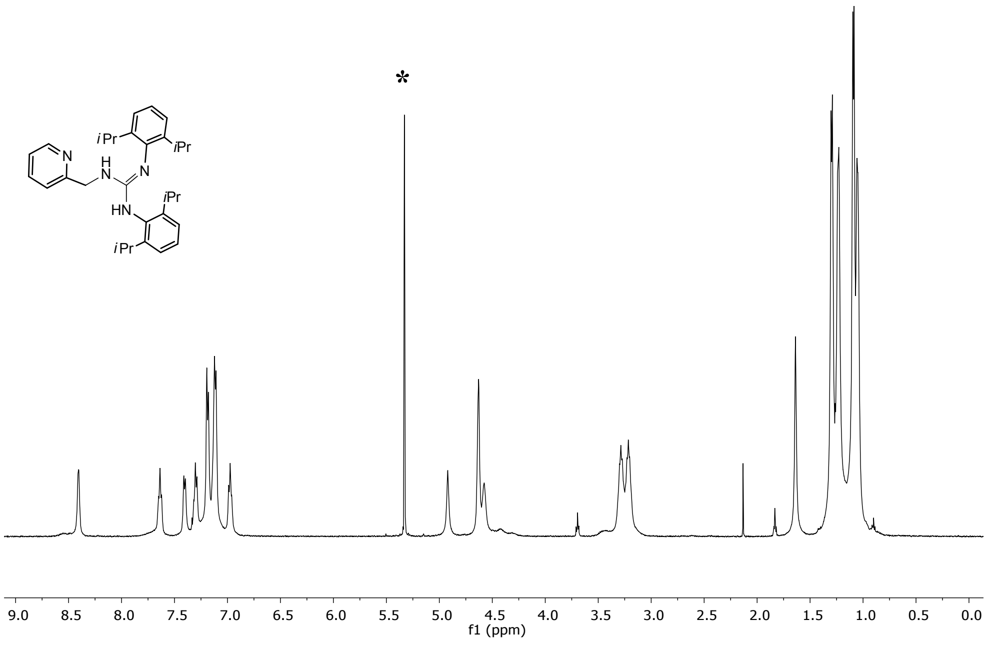

$^{13}C\{^{1}H\}$ NMR of HL1 (125.77 MHz, CD_2Cl_2, RT)

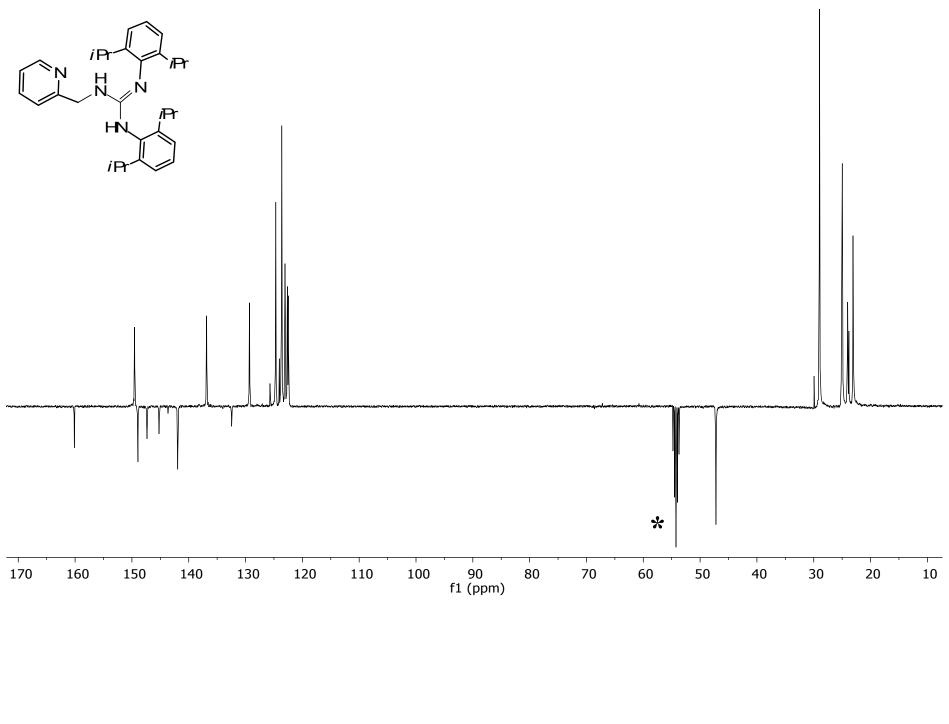

^{1}H NMR of HL2 (500.10 MHz, CD_2Cl_2, RT)

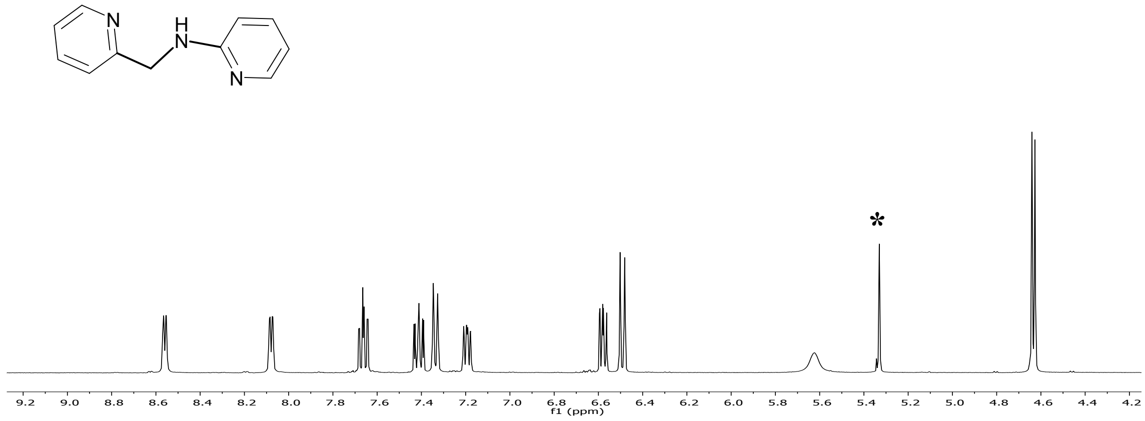

$^{13}C\{^{1}H\}$ NMR of HL2 (125.77 MHz, CD_2Cl_2, RT)

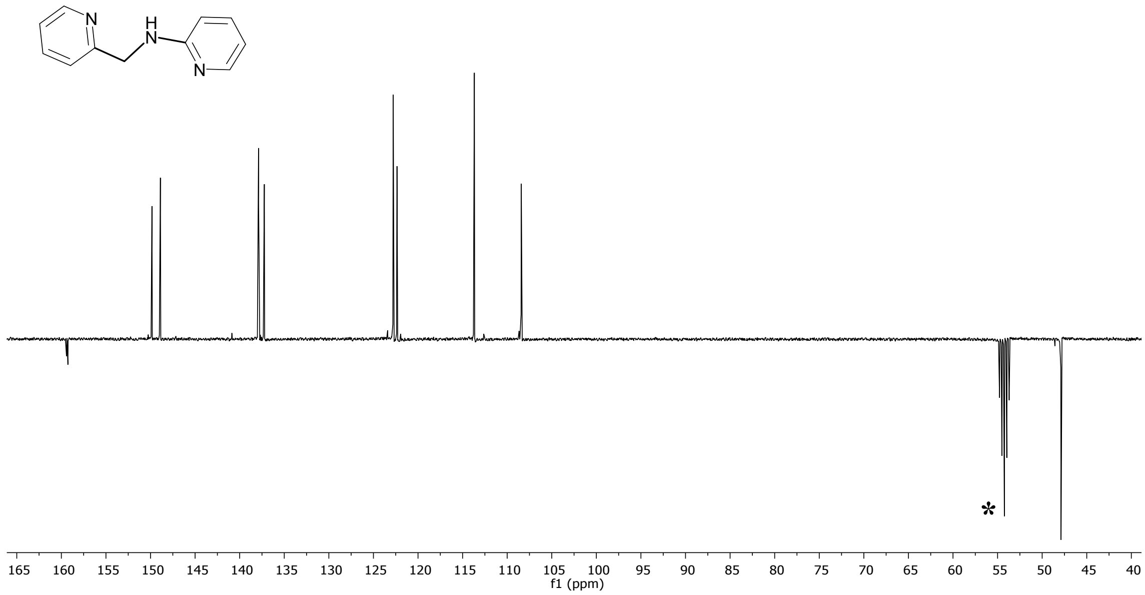

^{1}H NMR of [(Mes)Ru(κ^3*N*,*N'*,*N''*-L1)][SbF_6] (1) (500.10 MHz, CD_2Cl_2, RT)

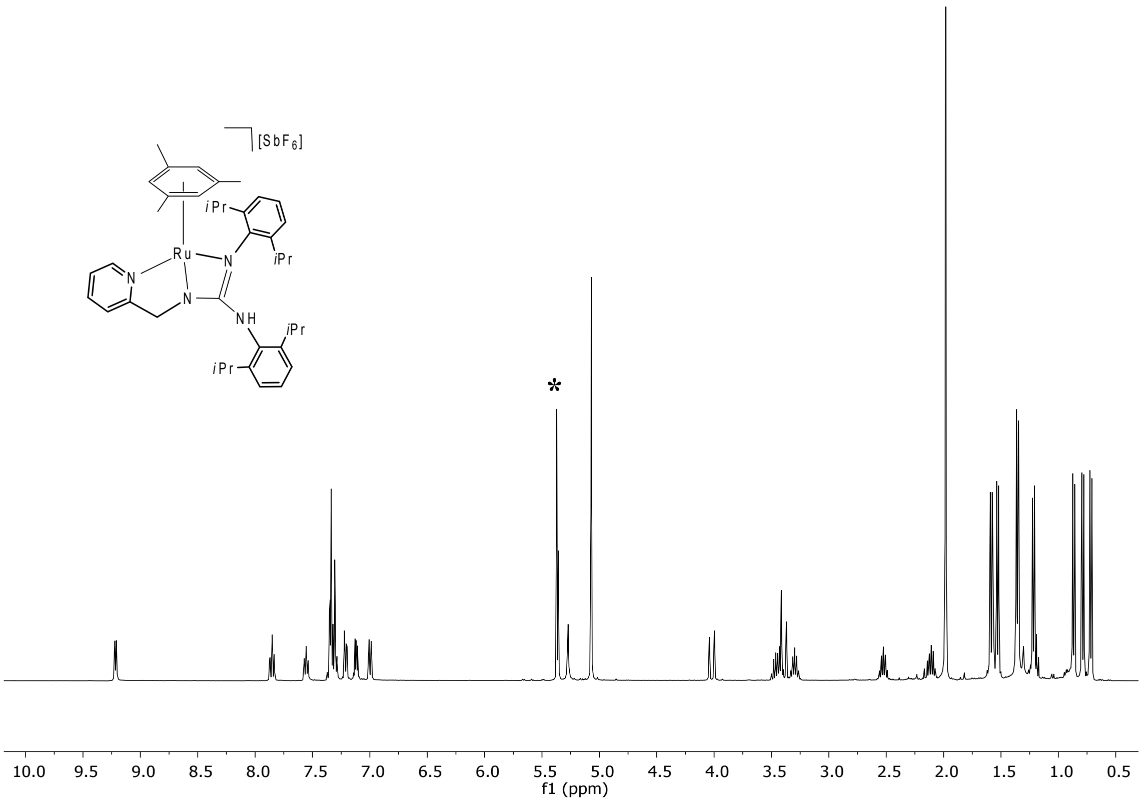

$^{13}C\{^{1}H\}$ NMR of [(Mes)Ru(κ^3*N*,*N'*,*N''*-L1)][SbF_6] (1) (125.77 MHz, CD_2Cl_2, RT)

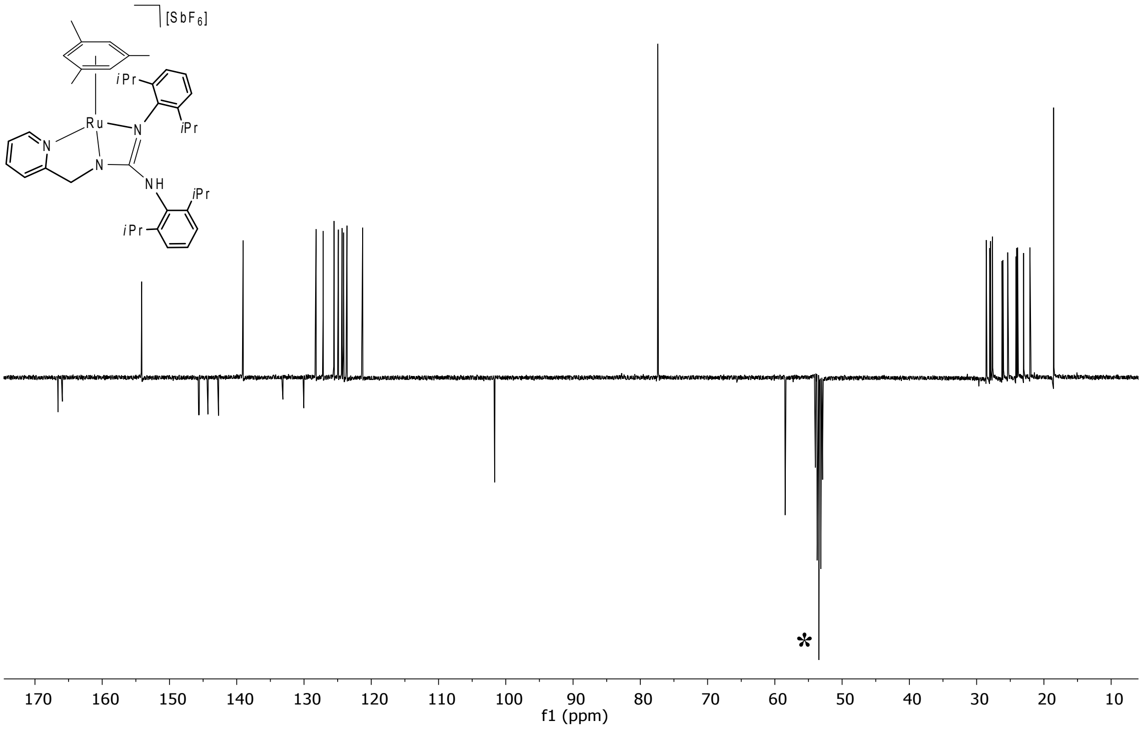

^{1}H NMR of [(Mes)RuCl(κ^{2}*N*,*N'*-HL1)][SbF$_6$] (2) (500.10 MHz, CD$_2$Cl$_2$, RT)

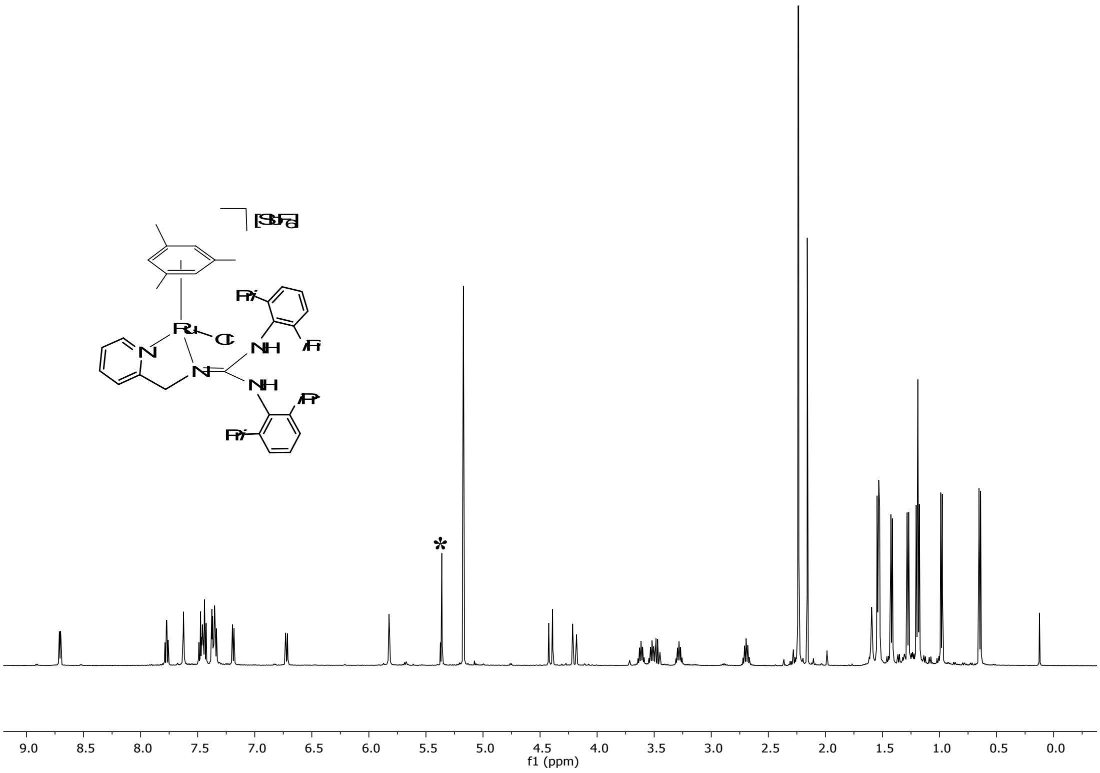

^{13}C{^{1}H} NMR of [(Mes)RuCl(κ^{2}*N*,*N'*-HL1)][SbF$_6$] (2) (125.77 MHz, CD$_2$Cl$_2$, RT)

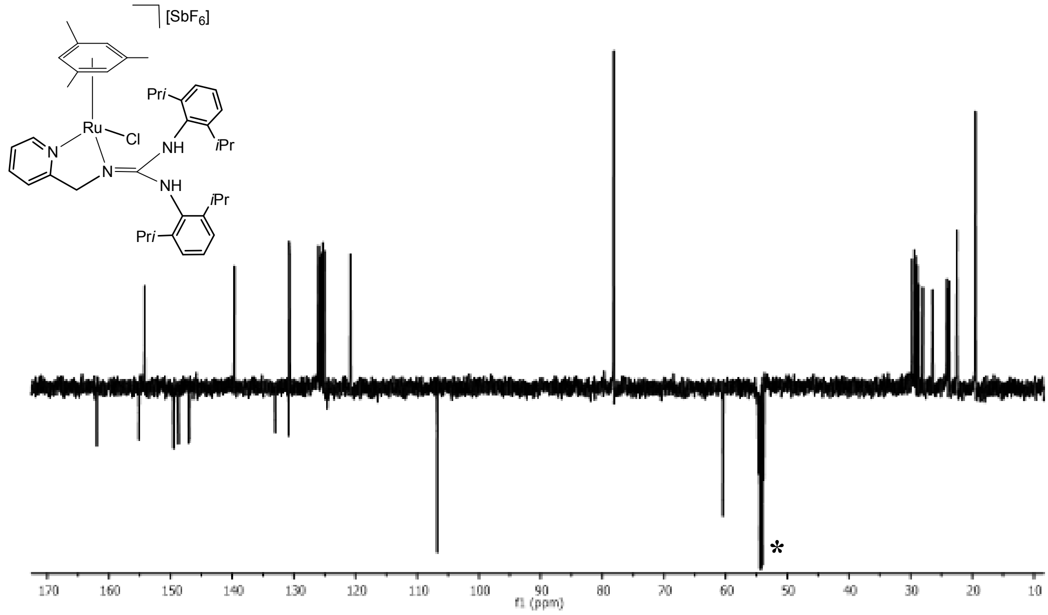

^{1}H NMR of a mixture of complexes 2, 1 and the guanidinium compound $H_2L1[SbF_6]$ in a 2/1/$H_2L1[SbF_6]$ molar ratio of 40/30/30 (500.10 MHz, CD_2Cl_2, RT)

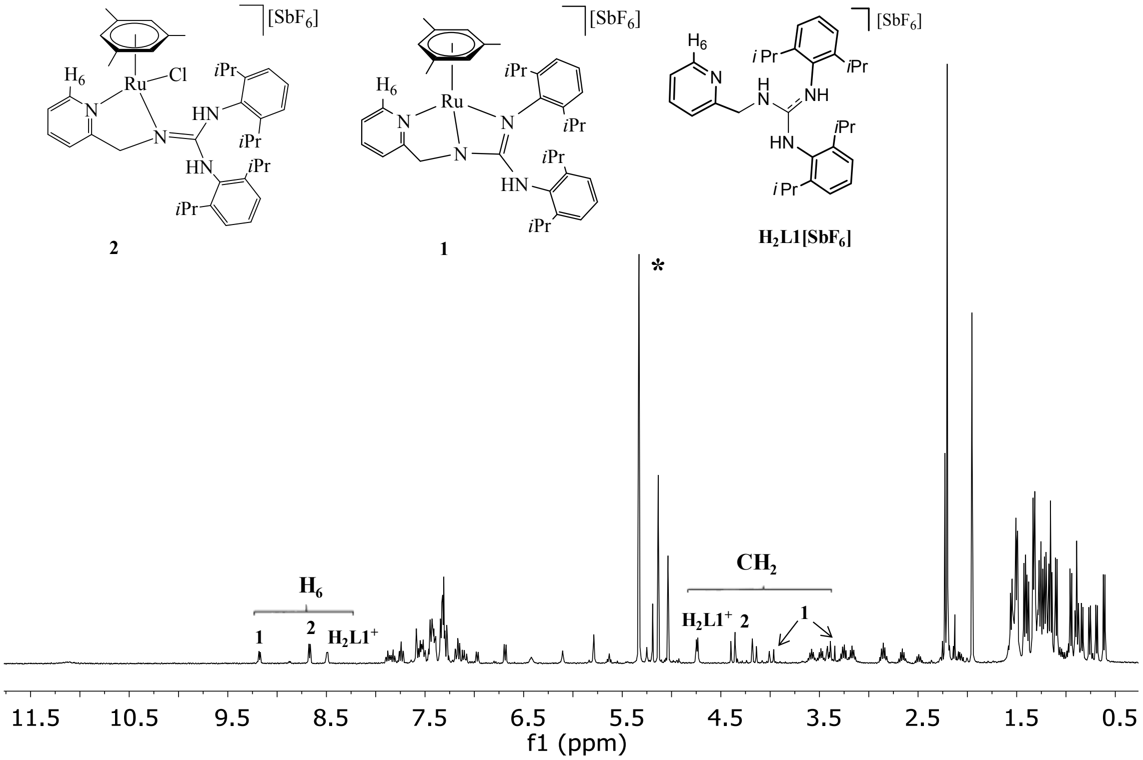

^{1}H NMR of [(Mes)RuCl(κ^{2}*N*,*N'*-HL2)][SbF_6] (3) (500.10 MHz, CD_2Cl_2, RT)

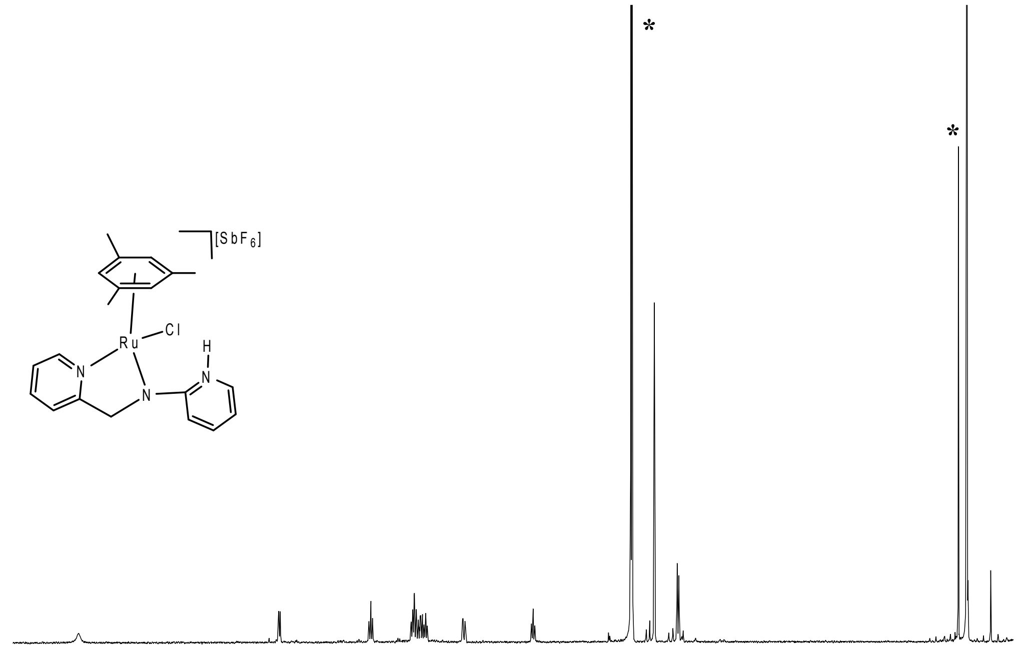

$^{13}C\{^{1}H\}$ NMR of [(Mes)RuCl(κ^{2}*N*,*N'*-HL2)][SbF_6] (3) (125.77 MHz, CD_2Cl_2, RT)

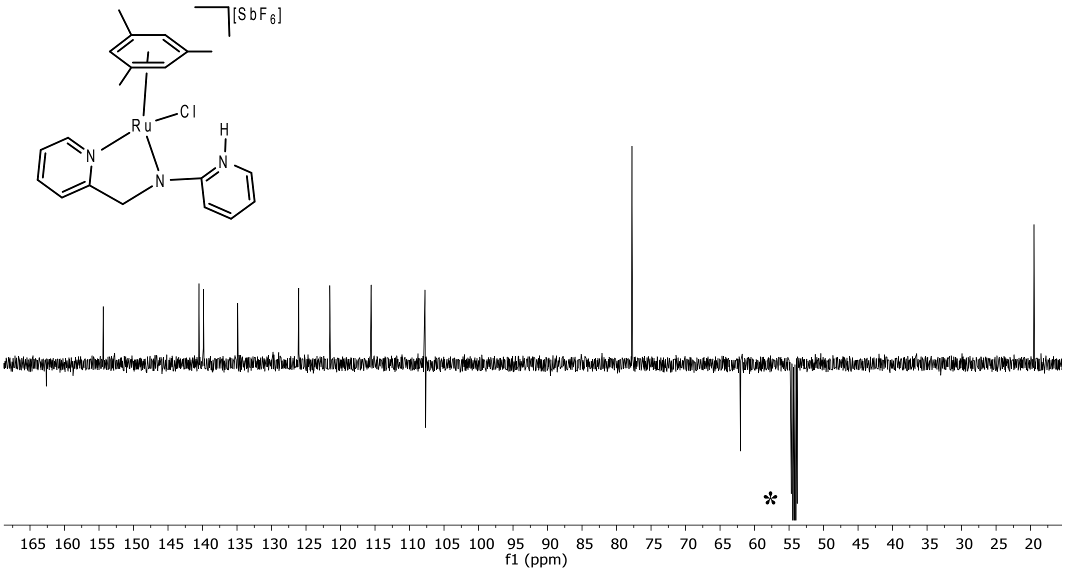

^{1}H NMR of [(Mes)Ru(κ^{3}*N*,*N'*,*N''*-L2)][SbF_6] (4) (500.10 MHz, CD_2Cl_2, RT)

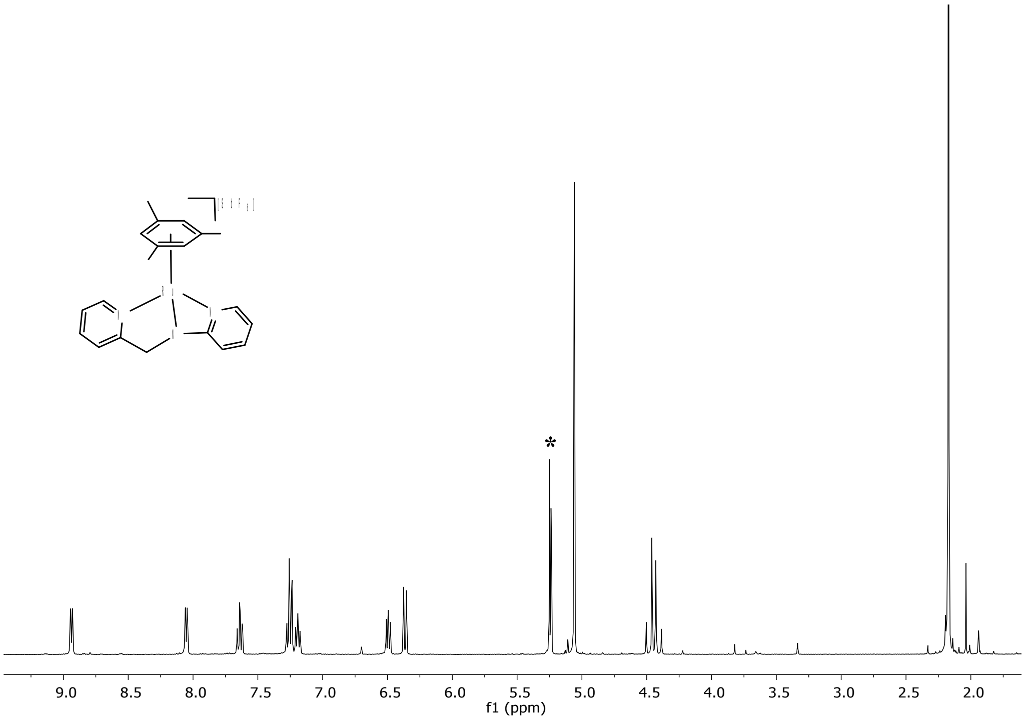

$^{13}C\{^{1}H\}$ NMR of [(Mes)Ru(κ^3*N,N',N"*-L2)][SbF_6] (4) (125.77 MHz, CD_2Cl_2, RT)

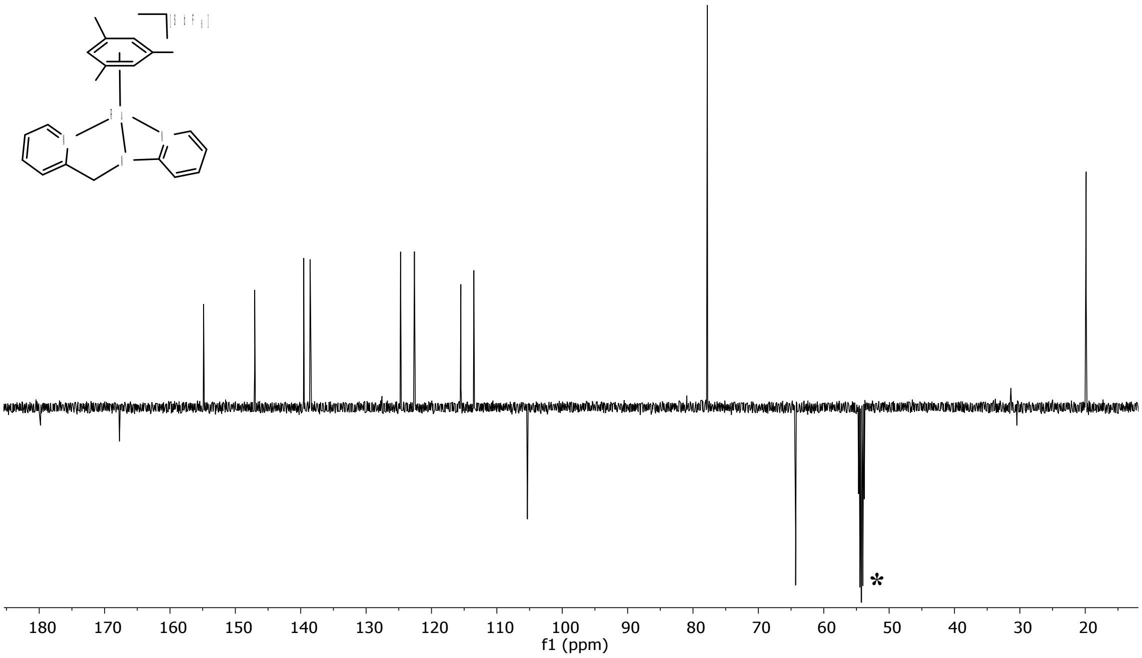

^{1}H NMR of [(Mes)Ru(κ^3*N,N',N"*-HL2)][SbF_6]$_2$ (5) (500.10 MHz, $(CD_3)_2CO$, RT)

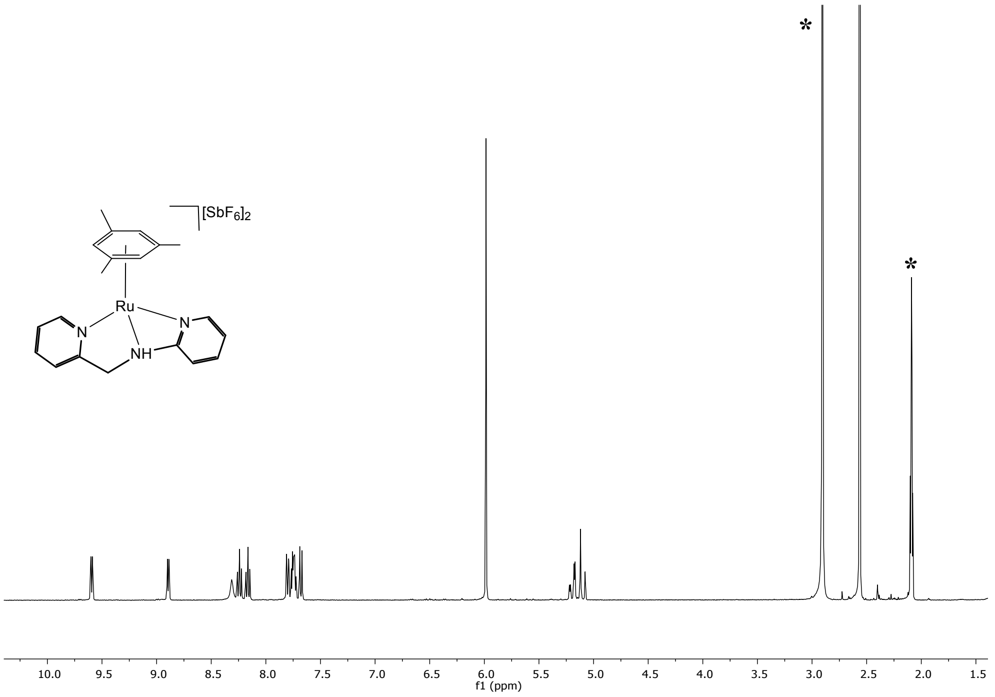

$^{13}C\{^{1}H\}$ NMR of [(Mes)Ru(κ^3*N*,*N'*,*N"*-HL2)][SbF$_6$]$_2$ (5) (125.77 MHz, CD$_3$)$_2$CO, RT)

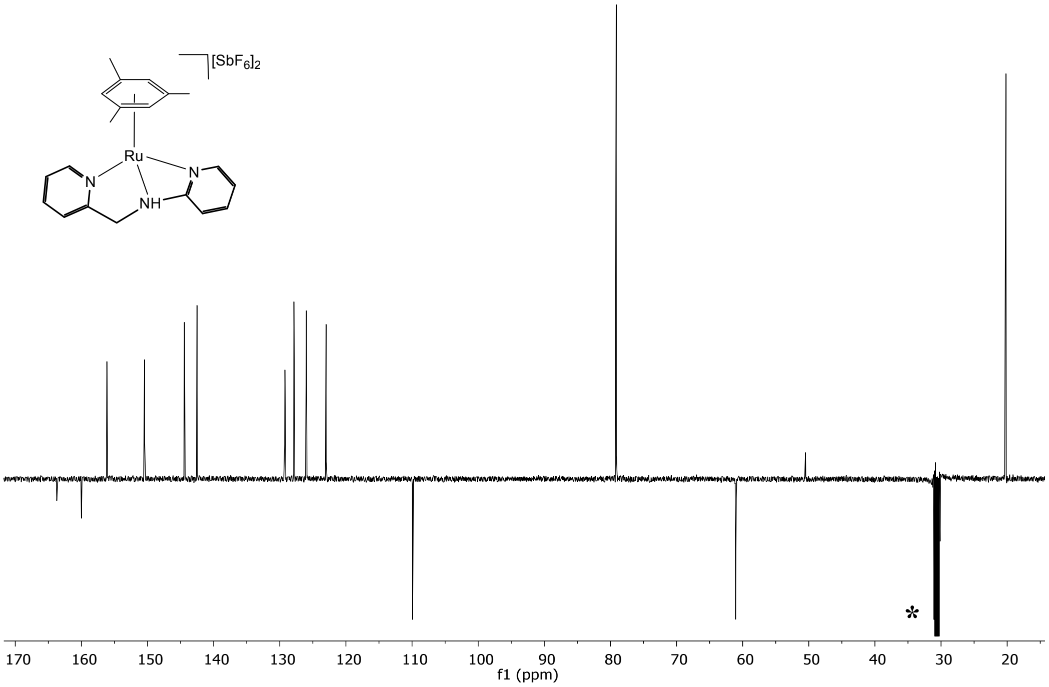

^{1}H NMR of a mixture of complexes 1/[(Mes)RuH(κ^2*N*,*N'*-HL1)][SbF$_6$] (6) in a 1/6 molar ratio of 98/2 (500.10 MHz, THF-d$_8$, RT)

The spectrum has been recorded during the formic acid dehydrogenation catalysis

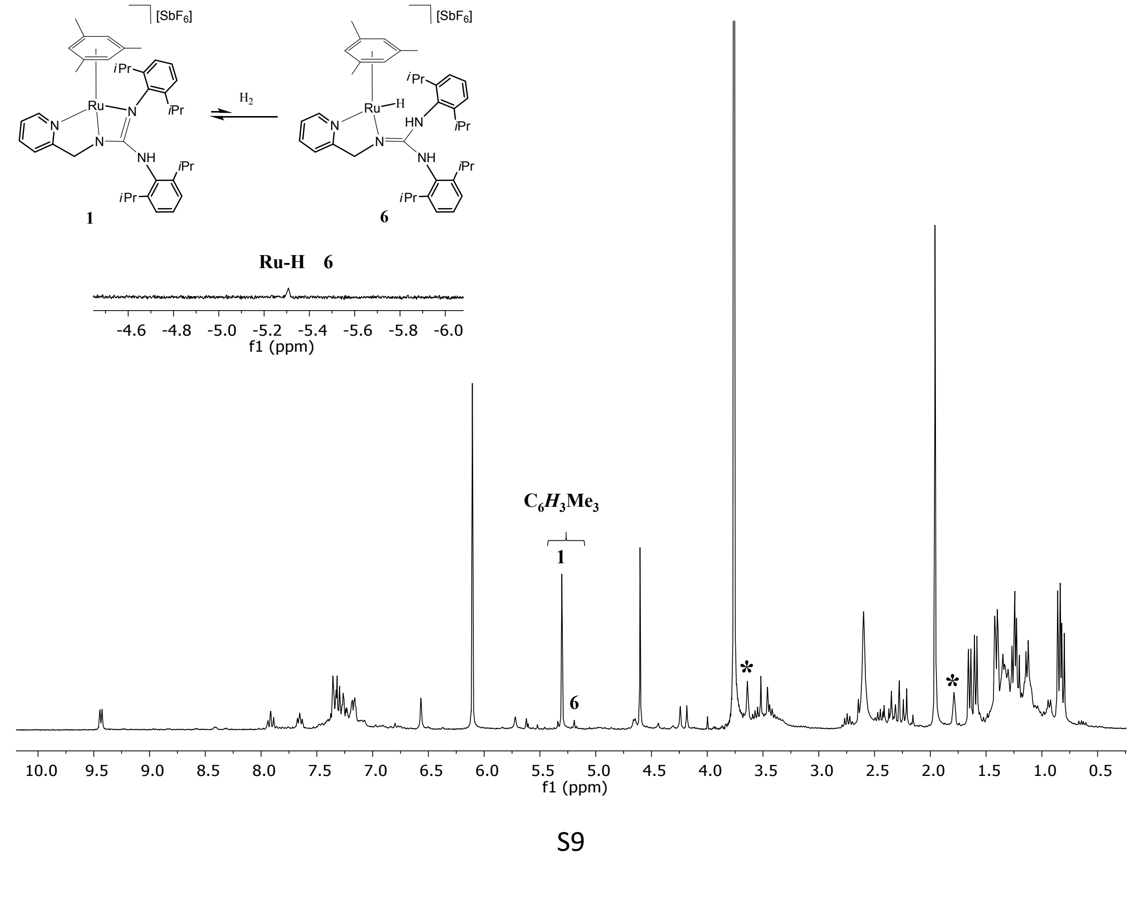

^{1}H NMR of a mixture of complexes 4/[(Mes)RuH(κ^2*N*,*N'*-HL2)][SbF$_6$] (7, 7') in a 4/7/7' molar ratio of 51/45/4 (500.10 MHz, THF-d$_8$, RT)

The spectrum has been recorded under H_2 pressure

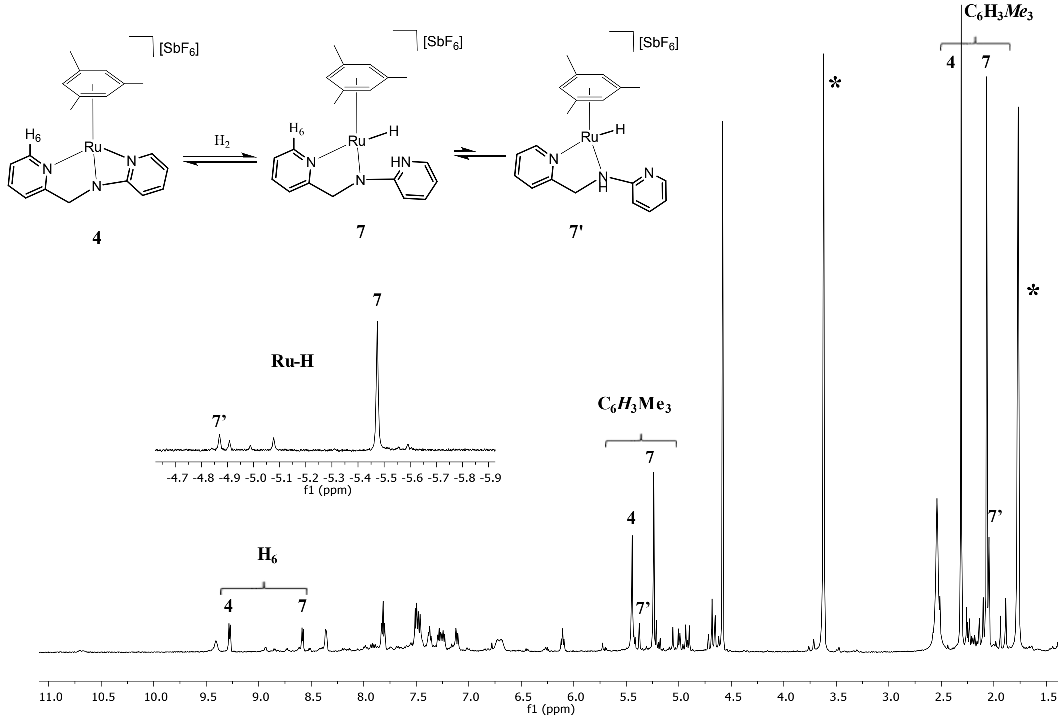

^{13}C{^{1}H} NMR of 3/[(Mes)RuH(κ^2*N*,*N'*-HL2)][SbF$_6$] (7, 7') in a 4/7/7' molar ratio of 51/45/4 (125.77 MHz, CD$_2$Cl$_2$, RT)

The spectrum has been recorded under H_2 pressure

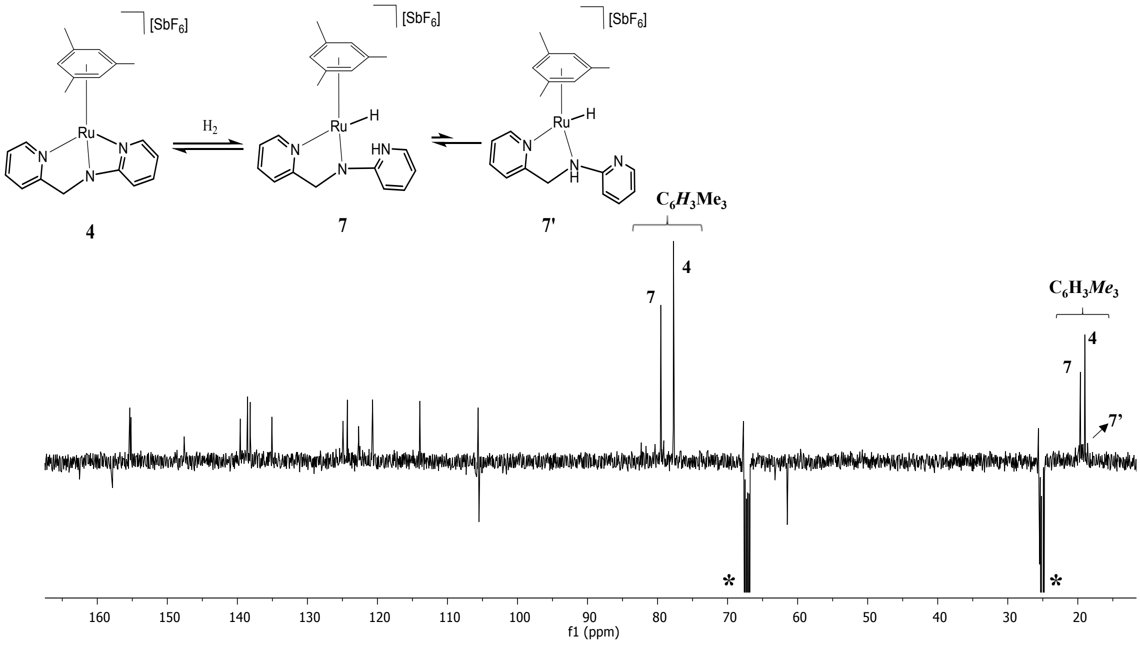

Selected region of the ^{1}H NMR where it is shown the presence of HD for complex 4 with H_2 at RT (500.10 MHz, THF-d_8 /D_2O, 0.45 mL/ 25µL, RT)

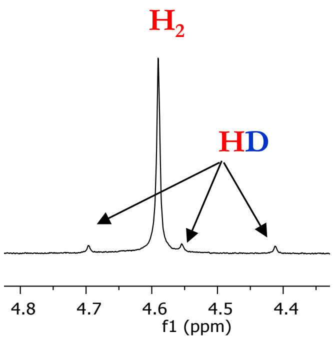

2 X-ray crystallography

The crystal structure of **HL1** and a selection of bond lengths and angles are given in Figure S1. As for the guanidine moiety, C9 exhibits a planar geometry [Σ^o_{C9} = 359.9(2)º], and one short carbon-nitrogen bond length [C9-N10 1.2889(16) Å] as well as two longer carbon-nitrogen bond lengths [C9-N8 1.3611(16), C9-N23 1.3819(16) Å] are observed indicating the presence of one formal double and two formal single carbon-nitrogen bonds, respectively. Accordingly, the H8 and H9 were observed in the Fourier difference map and could be refined freely.

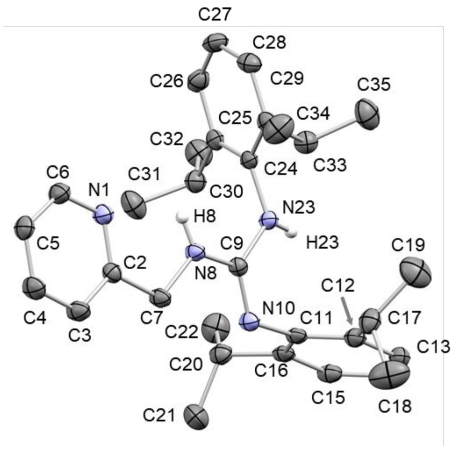

Figure S1. ORTEP view of **HL1**. Thermal ellipsoids are at 50 % probability. Most hydrogen atoms are omitted for clarity. Selected bond lengths (Å) and angles (˚) are: C9-N10 1.2889(16), C9-N8 1.3611(16), C9-N23 1.3819(16), N10-C9-N8 119.69(11), N10-C9-N23 124.13(11), N8-C9-N23 116.12(11).

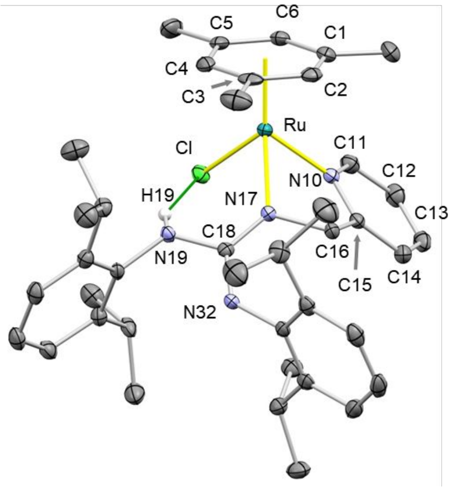

Figure S2. ORTEP view of the cation [(Mes)RuCl(κ^2*N,N'*-**HL1**)]$^+$ in **2**. Thermal ellipsoids are at 50 % probability. Most hydrogen atoms are omitted for clarity. Selected bond lengths (Å) and angles (˚) are: Ru-CT 1.699(4), N10-Ru 2.075(2), N17-Ru 2.144(2), Cl-Ru 2.3949(7), C18-N17 1.310(3), C18-N32 1.364(4), C18-N19 1.375(3), N10-Ru-CT 128.89(14), N17-Ru-CT 131.78(14), Cl-Ru-CT 128.61(11), N10-Ru-Cl 84.02(7), N17-Ru-Cl 89.05(7), N10-Ru-N17 76.48(9), N17-C18-N32 127.4(2), N17-C18-N19 118.3(2), N32-C18-N19 114.3(2), C18-N17-C16 122.5(2), C18-N17-Ru 130.92(19), C16-N17-Ru 105.96(16); N19-H19 0.813(36), N19···Cl 3.158(3), H19···Cl 2.407(36), N19-H19-Cl 154(3). CT, centroid of C15, C16, C17, C18, C19, and C20.

Single crystals of **3** were prepared by diffusion of hexane into a dichloromethane solution of the compound. Two sets of data were collected using different crystals, but in both cases, the quality of the data and the inherent crystal structure (inversion twinning and unclear systematic absence patterns) prevented a fully satisfactory crystal structure determination. Nonetheless, when the structure was preliminarily solved in the P1 space group (see File .res of **3** on page S15), a chemically meaningful picture could be obtained. Figure S3 shows a view of the unit cell that contains four independent cations [(Mes)RuCl(κ^2*N,N'*-**HL2**)]$^+$ and four independent anions SbF_6^-. Notably, even if the four cations are chemically equivalent, three of them exhibit an *R* configuration at the metal centre, whereas the remaining cation exhibits a positionally disordered **HL2** ligand [occupancy factors 0.52(2)/0.48(2)], rendering the *R* and *S* enantiomers. Also, it is worth mentioning that the structure was refined as an inversion twin with a Batch Scale Factor (BASF) converging to 0.26(6).

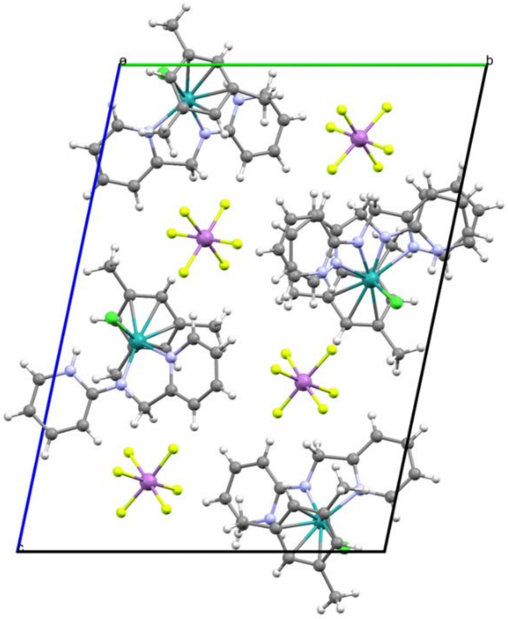

Figure S3. View along *a* of the unit cell of **3**.

Figure S4 shows a ball and stick view of the cation containing the atom Ru(1), as representative of the three *R* cations, along with the disordered cation. In both cases, the η^6–mesitylene ligand formally occupies three coordination sites, the ligand **HL2** binds in a κ^2*N,N'* fashion, and the remaining site is occupied by the chlorido ligand, thus defining a three-legged piano stool structure.

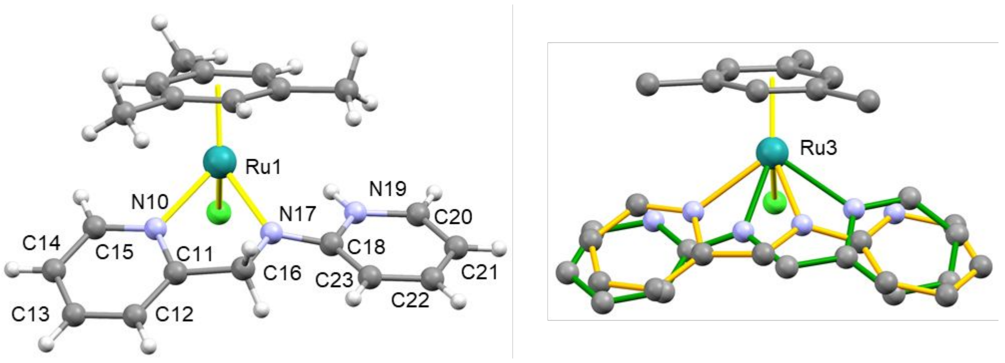

Figure S4. Views of independent cations [(Mes)RuCl(κ^2*N,N'*-**HL2**)]$^+$ in **3**. The components of the positionally disordered ligand **HL2** are given in orange and green.

Notably the planar geometry at the nitrogen atom N17 (Σ_{N17} = 359.0º), the relatively short C18–N17 distance (1.35 Å), and the small dihedral angle between the Ru–N17–C16–C18 plane and the adjacent pyridine ring (17.8º) nicely fit in with the proposed structure for **3** and, as discussed in the manuscript, confirms that the neutral resonance structure **II** should be the most relevant (Figure S5).[1]

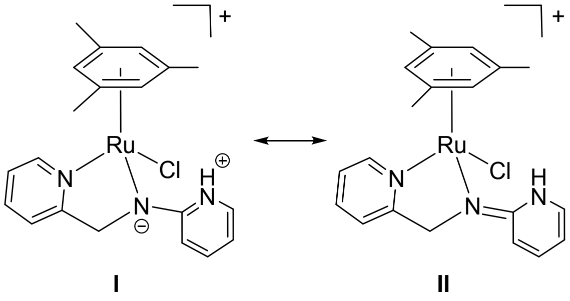

Figure S5. Resonance structures of **3**.

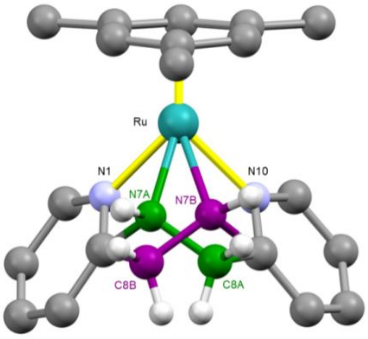

Figure S6. Ball-and-stick view of the crystal structure of the cation [(Mes)Ru(κ^3*N,N',N''*-**HL2**)]$^{2+}$ in **5**. For clarity, most hydrogen atoms are omitted and components of the positional disorder of the -CH_2-NH- linking moiety are shown in different colours, namely purple and green.

3. File .res of 3

The preliminary refinement of the crystal structure of compound **3** was performed using the SHELXL-2014[2] program within the WinGX suite.[3] The output file mo_VPX186_II_0sa_a.res is provided below.

```
TITL mo_VPX186_II_0sa_a.res in P1
    shelx.res
    created by SHELXL-2025/1 at 18:59:33 on 04-Feb-2026
CELL  0.71073   7.3975  15.4246  20.9012  101.880   90.058   90.008
ZERR     1.00   0.0007   0.0014   0.0018    0.001    0.001    0.001
LATT  -1
SFAC  C    H    N    F    CL   RU   SB
UNIT  80 92 12 24 4 4 4
MERG   2
TWIN
SADI 0.02 C1 C7 C3 C8 C5 C9
SADI 0.02 C7 C8 C8 C9 C9 C7
SADI 0.02 C51 C57 C53 C58 C55 C59
SADI 0.02 C57 C58 C58 C59 C59 C57
SADI 0.02 C101 C107 C103 C108 C105 C109
SADI 0.02 C107 C108 C108 C109 C109 C107
SADI 0.02 C151 C157 C153 C158 C155 C159
SADI 0.02 C157 C158 C158 C159 C159 C157
SADI  0.02 SB1 F1 SB1 F2 SB1 F3 SB1 F5 SB1 F5 SB1 F6
SADI  0.02 SB2 F7 SB2 F8 SB2 F9 SB2 F10 SB2 F12 SB2 F12
SADI  0.02 SB3 F13 SB3 F14 SB3 F17 SB3 F18 SB3 F17 SB3 F18
SADI  0.02 SB4 F19 SB4 F20 SB4 F21 SB4 F22 SB4 F24 SB4 F24
DFIX 31.00 0.01 F1 F2  F1 F3  F1 F4  F1 F5 F6 F2  F6 F3
DFIX 31.00 0.01 F6 F4  F6 F5  F2 F3  F2 F5 F4 F3  F4 F5
DFIX 31.00 0.01 F7 F8  F7 F9  F7 F10  F7 F11 F12 F8  F12 F9
DFIX 31.00 0.01 F12 F10  F12 F11  F8 F9  F8 F11 F10 F9  F10 F11
DFIX 31.00 0.01 F13 F14  F13 F15  F13 F16  F13 F17  F18 F14  F18
F15
DFIX 31.00 0.01 F18 F16  F18 F17  F14 F15  F14 F17  F16 F15  F16
F17
DFIX 31.00 0.01 F19 F20  F19 F21  F19 F22  F19 F23  F24 F20  F24
F21
DFIX 31.00 0.01 F24 F22  F24 F23  F20 F21  F20 F23  F22 F21  F22
F23
FMAP   2
PLAN   20
ACTA    50.00
BOND   $H
CONF
L.S.  10
TEMP  -173.00
WGHT    0.077700   30.744701
BASF   0.26272
FVAR       0.25600   0.51722   2.59341
MOLE  1
AFIX  66
C1    1    0.284783   -0.169482    1.028324    11.00000    0.05189
C2    1    0.189373   -0.133858    0.982517    11.00000    0.03337
AFIX  43
H2    2    0.150152   -0.073965    0.992986    11.00000   -1.20000
AFIX  65
C3    1    0.151351   -0.185867    0.921393    11.00000    0.03124
```

```
C4    1    0.208738   -0.273500    0.906075    11.00000    0.04649
AFIX  43
H4    2    0.182750   -0.309045    0.864299    11.00000   -1.20000
AFIX  65
C5    1    0.304148   -0.309125    0.951882    11.00000    0.05058
C6    1    0.342171   -0.257117    1.013006    11.00000    0.05614
AFIX  43
H6    2    0.407380   -0.281465    1.044313    11.00000   -1.20000
AFIX   0
C7    1    0.333903   -0.107202    1.093721    11.00000    0.07810
AFIX 137
H7A   2    0.223025   -0.087076    1.117876    11.00000   -1.50000
H7B   2    0.400399   -0.055882    1.085062    11.00000   -1.50000
H7C   2    0.409478   -0.139099    1.119756    11.00000   -1.50000
AFIX   0
C8    1    0.053237   -0.148527    0.869437    11.00000    0.04538
AFIX 137
H8A   2   -0.077508   -0.154454    0.874730    11.00000   -1.50000
H8B   2    0.089473   -0.181175    0.826048    11.00000   -1.50000
H8C   2    0.084259   -0.085792    0.873915    11.00000   -1.50000
AFIX   0
C9    1    0.367737   -0.404499    0.939295    11.00000    0.07805
AFIX 137
H9A   2    0.274973   -0.441377    0.953952    11.00000   -1.50000
H9B   2    0.480179   -0.408319    0.963392    11.00000   -1.50000
H9C   2    0.389189   -0.425487    0.892400    11.00000   -1.50000
AFIX  66
N10   3    0.471396   -0.101565    0.872793    11.00000    0.02111
C11   1    0.534183   -0.138472    0.810481    11.00000    0.02985
C12   1    0.543841   -0.087087    0.763209    11.00000    0.04726
AFIX  43
H12   2    0.586753   -0.112312    0.720621    11.00000   -1.20000
AFIX  65
C13   1    0.490713    0.001204    0.778248    11.00000    0.05559
AFIX  43
H13   2    0.497314    0.036323    0.745939    11.00000   -1.20000
AFIX  65
C14   1    0.427925    0.038112    0.840560    11.00000    0.03943
AFIX  43
H14   2    0.391615    0.098455    0.850839    11.00000   -1.20000
AFIX  65
C15   1    0.418266   -0.013271    0.887833    11.00000    0.03625
AFIX  43
H15   2    0.375354    0.011954    0.930421    11.00000   -1.20000
AFIX   0
C16   1    0.589817   -0.234262    0.803292    11.00000    0.04620
AFIX  23
H16A  2    0.494600   -0.273448    0.779764    11.00000   -1.20000
H16B  2    0.703026   -0.245284    0.777689    11.00000   -1.20000
AFIX   0
N17   3    0.616573   -0.252923    0.866539    11.00000    0.02421
AFIX  66
C18   1    0.731228   -0.320190    0.869723    11.00000    0.03544
N19   3    0.766276   -0.330017    0.933191    11.00000    0.03307
AFIX  43
H19   2    0.725079   -0.290692    0.966596    11.00000   -1.20000
AFIX  65
C20   1    0.866395   -0.401959    0.943896    11.00000    0.03350
```

```
AFIX  43
H20   2    0.890348   -0.408675    0.987274    11.00000   -1.20000
AFIX  65
C21   1    0.931467   -0.464074    0.891131    11.00000    0.04818
AFIX  43
H21   2    0.999895   -0.513243    0.898448    11.00000   -1.20000
AFIX  65
C22   1    0.896420   -0.454248    0.827663    11.00000    0.04852
AFIX  43
H22   2    0.940895   -0.496701    0.791601    11.00000   -1.20000
AFIX  65
C23   1    0.796301   -0.382307    0.816957    11.00000    0.04465
AFIX  43
H23   2    0.772348   -0.375591    0.773579    11.00000   -1.20000
AFIX   0
CL1   5    0.698286   -0.111877    0.992485    11.00000    0.03683
0.03875 =
         0.03521   -0.00771   -0.01053    0.00073
RU1   6    0.442129   -0.189098    0.937009    11.00000    0.02277
0.03118 =
         0.03336    0.00167    0.00952   -0.00514
MOLE  2
AFIX  66
C51   1    0.160086    0.159427    0.473461    11.00000    0.04207
C52   1    0.110252    0.247197    0.477128    11.00000    0.04767
AFIX  43
H52   2    0.045951    0.263520    0.442050    11.00000   -1.20000
AFIX  65
C53   1    0.154499    0.311085    0.532118    11.00000    0.04133
C54   1    0.248580    0.287203    0.583442    11.00000    0.05098
AFIX  43
H54   2    0.278821    0.330868    0.621026    11.00000   -1.20000
AFIX  65
C55   1    0.298415    0.199433    0.579777    11.00000    0.04061
C56   1    0.254170    0.135544    0.524786    11.00000    0.04220
AFIX  43
H56   2    0.288230    0.075558    0.522281    11.00000   -1.20000
AFIX   0
C58   1    0.109470    0.409089    0.537292    11.00000    0.08469
AFIX 137
H58A  2   -0.018604    0.415201    0.527034    11.00000   -1.50000
H58B  2    0.184008    0.433131    0.506311    11.00000   -1.50000
H58C  2    0.134161    0.441711    0.581844    11.00000   -1.50000
AFIX   0
C57   1    0.095243    0.088991    0.414106    11.00000    0.07391
AFIX 137
H57A  2   -0.030969    0.100614    0.404325    11.00000   -1.50000
H57B  2    0.104950    0.029944    0.424328    11.00000   -1.50000
H57C  2    0.170670    0.091793    0.376067    11.00000   -1.50000
AFIX   0
C59   1    0.393109    0.167141    0.636934    11.00000    0.06490
AFIX 137
H59A  2    0.328797    0.115463    0.646036    11.00000   -1.50000
H59B  2    0.391950    0.214760    0.676037    11.00000   -1.50000
H59C  2    0.518363    0.150811    0.624817    11.00000   -1.50000
AFIX  66
N60   3   -0.202139    0.307102    0.601243    11.00000    0.05404
C61   1   -0.224481    0.326804    0.668707    11.00000    0.06023
```

```
C62   1   -0.334385    0.397034    0.697665    11.00000    0.04360
AFIX  43
H62   2   -0.349655    0.410500    0.743774    11.00000   -1.20000
AFIX  65
C63   1   -0.421948    0.447562    0.659158    11.00000    0.06773
AFIX  43
H63   2   -0.497062    0.495561    0.678950    11.00000   -1.20000
AFIX  65
C64   1   -0.399607    0.427860    0.591694    11.00000    0.04467
AFIX  43
H64   2   -0.459453    0.462393    0.565376    11.00000   -1.20000
AFIX  65
C65   1   -0.289704    0.357630    0.562735    11.00000    0.05263
AFIX  43
H65   2   -0.274435    0.344165    0.516626    11.00000   -1.20000
AFIX   0
C66   1   -0.117587    0.263613    0.704499    11.00000    0.05090
AFIX  23
H66A  2   -0.010848    0.294737    0.726922    11.00000   -1.20000
H66B  2   -0.195528    0.245075    0.737760    11.00000   -1.20000
AFIX   0
N67   3   -0.060568    0.187115    0.657059    11.00000    0.06724
AFIX  66
C68   1   -0.038285    0.113651    0.676109    11.00000    0.05238
N69   3    0.014233    0.040822    0.628867    11.00000    0.04732
AFIX  43
H69   2    0.026370    0.045833    0.587905    11.00000   -1.20000
AFIX  65
C70   1    0.047580   -0.039921    0.646323    11.00000    0.04222
AFIX  43
H70   2    0.083474   -0.089696    0.614035    11.00000   -1.20000
AFIX  65
C71   1    0.028410   -0.047836    0.711023    11.00000    0.08953
AFIX  43
H71   2    0.051202   -0.103020    0.722954    11.00000   -1.20000
AFIX  65
C72   1   -0.024108    0.024993    0.758266    11.00000    0.06712
AFIX  43
H72   2   -0.037210    0.019583    0.802486    11.00000   -1.20000
AFIX  65
C73   1   -0.057456    0.105737    0.740810    11.00000    0.06486
AFIX  43
H73   2   -0.093350    0.155512    0.773099    11.00000   -1.20000
AFIX   0
RU2   6    0.013851    0.206760    0.568054    11.00000    0.04747
0.04563 =
         0.04523   -0.01137    0.02393   -0.02188
CL2   5   -0.241293    0.122417    0.520009    11.00000    0.05630
0.04198 =
         0.03371   -0.00387   -0.00592   -0.00137
MOLE  3
C107  1    0.592345    0.924848    0.594247    11.00000    0.06118
AFIX 137
H10A  2    0.473686    0.907632    0.608337    11.00000   -1.50000
H10B  2    0.582497    0.982094    0.581100    11.00000   -1.50000
H10C  2    0.678578    0.929790    0.630445    11.00000   -1.50000
AFIX   0
C109  1    0.602606    0.601942    0.469666    11.00000    0.07709
```

```
AFIX 137
H10D  2    0.575877    0.576624    0.423683    11.00000   -1.50000
H10E  2    0.495738    0.596880    0.496328    11.00000   -1.50000
H10F  2    0.703617    0.569739    0.484274    11.00000   -1.50000
PART  1
AFIX  66
N610  3    0.300968    0.703314    0.407756    21.00000    0.01459
C611  1    0.278295    0.684860    0.340286    21.00000    0.01852
C612  1    0.167741    0.615169    0.310682    21.00000    0.02292
AFIX  43
H612  2    0.152246    0.602557    0.264569    21.00000   -1.20000
AFIX  65
C613  1    0.079860    0.563932    0.348549    21.00000    0.02652
AFIX  43
H613  2    0.004301    0.516301    0.328316    21.00000   -1.20000
AFIX  65
C614  1    0.102531    0.582385    0.416019    21.00000    0.02533
AFIX  43
H614  2    0.042468    0.547366    0.441899    21.00000   -1.20000
AFIX  65
C615  1    0.213085    0.652076    0.445623    21.00000    0.03382
AFIX  43
H615  2    0.228580    0.664687    0.491737    21.00000   -1.20000
AFIX   0
C616  1    0.387060    0.742558    0.306639    21.00000    0.02099
AFIX  23
H61A  2    0.496976    0.710821    0.287566    21.00000   -1.20000
H61B  2    0.315758    0.759014    0.270835    21.00000   -1.20000
AFIX   0
N617  3    0.437096    0.821591    0.354169    21.00000    0.01122
AFIX  66
C618  1    0.462675    0.898902    0.333047    21.00000    0.01936
C623  1    0.454151    0.906249    0.267938    21.00000    0.04566
AFIX  43
H623  2    0.424330    0.855993    0.235042    21.00000   -1.20000
AFIX  65
C622  1    0.489260    0.987128    0.250959    21.00000    0.03268
AFIX  43
H622  2    0.483435    0.992150    0.206460    21.00000   -1.20000
AFIX  65
C621  1    0.532894    1.060661    0.299090    21.00000    0.03333
AFIX  43
H621  2    0.556890    1.115938    0.287486    21.00000   -1.20000
AFIX  65
C620  1    0.541418    1.053315    0.364199    21.00000    0.01441
AFIX  43
H620  2    0.571240    1.103571    0.397094    21.00000   -1.20000
AFIX  65
N619  3    0.506309    0.972436    0.381178    21.00000    0.01923
AFIX  43
H619  2    0.511706    0.967785    0.422399    21.00000   -1.20000
PART  2
AFIX  66
C111  1    0.433816    0.870254    0.312278   -21.00000    0.01841
C112  1    0.424796    0.923551    0.266234   -21.00000    0.02546
AFIX  43
H112  2    0.380530    0.899938    0.223518   -21.00000   -1.20000
AFIX  65
```

```
C113  1    0.480543    1.011399    0.282691   -21.00000    0.04131
AFIX  43
H113  2    0.474377    1.047825    0.251221   -21.00000   -1.20000
AFIX  65
C114  1    0.545310    1.045949    0.345191   -21.00000    0.04218
AFIX  43
H114  2    0.583410    1.105989    0.356438   -21.00000   -1.20000
AFIX  65
C115  1    0.554331    0.992652    0.391235   -21.00000    0.04162
AFIX  43
H115  2    0.598597    1.016266    0.433951   -21.00000   -1.20000
AFIX  65
N110  3    0.498585    0.904805    0.374780   -21.00000    0.01044
AFIX  66
N119  3    0.203893    0.669539    0.426618   -21.00000    0.02756
AFIX  43
H119  2    0.246944    0.706147    0.461209   -21.00000   -1.20000
AFIX  65
C118  1    0.236675    0.685018    0.364458   -21.00000    0.02144
C123  1    0.168675    0.627194    0.309819   -21.00000    0.05605
AFIX  43
H123  2    0.191080    0.637773    0.267336   -21.00000   -1.20000
AFIX  65
C122  1    0.067892    0.553892    0.317339   -21.00000    0.03980
AFIX  43
H122  2    0.021417    0.514373    0.279995   -21.00000   -1.20000
AFIX  65
C121  1    0.035109    0.538413    0.379498   -21.00000    0.05670
AFIX  43
H121  2   -0.033772    0.488314    0.384637   -21.00000   -1.20000
AFIX  65
C120  1    0.103108    0.596236    0.434138   -21.00000    0.03259
AFIX  43
H120  2    0.080702    0.585656    0.476621   -21.00000   -1.20000
AFIX   0
C116  1    0.375778    0.778187    0.298179   -21.00000    0.02310
AFIX  23
H11A  2    0.468791    0.740351    0.272152   -21.00000   -1.20000
H11B  2    0.261456    0.771719    0.272975   -21.00000   -1.20000
AFIX   0
N117  3    0.347861    0.750618    0.364707   -21.00000    0.01766
PART  0
AFIX  66
C102  1    0.751766    0.876978    0.483422    11.00000    0.03945
AFIX  43
H102  2    0.785052    0.936996    0.485564    11.00000   -1.20000
AFIX  65
C103  1    0.795241    0.812823    0.428537    11.00000    0.03306
C104  1    0.746539    0.725008    0.425403    11.00000    0.06431
AFIX  43
H104  2    0.776253    0.681161    0.387892    11.00000   -1.20000
AFIX  65
C105  1    0.654362    0.701347    0.477154    11.00000    0.04785
C106  1    0.610886    0.765501    0.532039    11.00000    0.05364
AFIX  43
H106  2    0.547886    0.749329    0.567408    11.00000   -1.20000
AFIX  65
C101  1    0.659587    0.853316    0.535174    11.00000    0.03914
```

```
AFIX   0
C108  1    0.896400    0.843700    0.370824    11.00000    0.06389
AFIX 137
H10G  2    0.970655    0.895799    0.388400    11.00000   -1.50000
H10H  2    0.807500    0.858850    0.340102    11.00000   -1.50000
H10I  2    0.973994    0.795664    0.348057    11.00000   -1.50000
AFIX   0
CL3   5    0.258697    0.890016    0.488932    11.00000    0.05852
0.04124 =
         0.03756   -0.00648    0.01271   -0.00023
RU3   6    0.513702    0.806303    0.440979    11.00000    0.04817
0.04343 =
         0.04389   -0.01153   -0.02447    0.02019
MOLE  4
C151  1    0.786379    1.182317   -0.019690    11.00000    0.05097
C152  1    0.841731    1.264838   -0.005993    11.00000    0.05376
AFIX  43
H152  2    0.911258    1.285458   -0.038033    11.00000   -1.20000
AFIX   0
C153  1    0.807351    1.321318    0.049393    11.00000    0.04644
C154  1    0.709408    1.287513    0.101810    11.00000    0.04195
AFIX  43
H154  2    0.687817    1.325370    0.143049    11.00000   -1.20000
AFIX   0
C155  1    0.647585    1.196199    0.089054    11.00000    0.02988
C156  1    0.685294    1.145272    0.026058    11.00000    0.03100
AFIX  43
H156  2    0.642868    1.085987    0.014234    11.00000   -1.20000
AFIX   0
C157  1    0.837288    1.119944   -0.081210    11.00000    0.08214
AFIX 137
H15A  2    0.764919    1.065780   -0.085854    11.00000   -1.50000
H15B  2    0.965839    1.105119   -0.079756    11.00000   -1.50000
H15C  2    0.814646    1.147841   -0.118552    11.00000   -1.50000
AFIX   0
C158  1    0.859193    1.414755    0.071008    11.00000    0.12490
AFIX 137
H15D  2    0.966612    1.426959    0.046909    11.00000   -1.50000
H15E  2    0.886064    1.426885    0.117930    11.00000   -1.50000
H15F  2    0.759473    1.452729    0.062599    11.00000   -1.50000
AFIX   0
C159  1    0.552176    1.159629    0.138964    11.00000    0.05052
AFIX 137
H15G  2    0.573581    1.197456    0.182067    11.00000   -1.50000
H15H  2    0.596877    1.099746    0.138684    11.00000   -1.50000
H15I  2    0.422275    1.157232    0.129512    11.00000   -1.50000
AFIX   0
C166  1    1.089848    1.252288    0.205025    11.00000    0.05001
AFIX  23
H16C  2    0.993728    1.291463    0.227827    11.00000   -1.20000
H16D  2    1.202331    1.264787    0.230994    11.00000   -1.20000
AFIX   0
N167  3    1.118209    1.271173    0.140998    11.00000    0.05478
AFIX  66
N160  3    0.975849    1.117446    0.138379    11.00000    0.05143
C161  1    0.924374    1.028872    0.124891    11.00000    0.03934
AFIX  43
H161  2    0.883101    1.001918    0.082391    11.00000   -1.20000
```

```
AFIX  65
C162  1    0.933289    0.979737    0.173585    11.00000    0.05839
AFIX  43
H162  2    0.898109    0.919200    0.164366    11.00000   -1.20000
AFIX  65
C163  1    0.993679    1.019176    0.235769    11.00000    0.08596
AFIX  43
H163  2    0.999771    0.985593    0.269049    11.00000   -1.20000
AFIX  65
C164  1    1.045154    1.107749    0.249258    11.00000    0.05524
AFIX  43
H164  2    1.086427    1.134703    0.291758    11.00000   -1.20000
AFIX  65
C165  1    1.036240    1.156886    0.200564    11.00000    0.06214
AFIX  66
C168  1    1.229243    1.335313    0.135952    11.00000    0.04964
N169  3    1.265784    1.343925    0.072337    11.00000    0.04381
AFIX  43
H169  2    1.224914    1.304163    0.039094    11.00000   -1.20000
AFIX  65
C170  1    1.366881    1.415342    0.061230    11.00000    0.05616
AFIX  43
H170  2    1.391855    1.421228    0.017752    11.00000   -1.20000
AFIX  65
C171  1    1.431437    1.478148    0.113738    11.00000    0.07170
AFIX  43
H171  2    1.500533    1.526958    0.106147    11.00000   -1.20000
AFIX  65
C172  1    1.394897    1.469537    0.177354    11.00000    0.06741
AFIX  43
H172  2    1.439019    1.512462    0.213241    11.00000   -1.20000
AFIX  65
C173  1    1.293801    1.398120    0.188462    11.00000    0.04881
AFIX  43
H173  2    1.268828    1.392234    0.231940    11.00000   -1.20000
AFIX   0
CL4   5    1.198180    1.123706    0.017408    11.00000    0.03505
0.03875 =
         0.03614   -0.00600    0.00781   -0.00070
RU4   6    0.942261    1.202375    0.071815    11.00000    0.02255
0.03259 =
         0.03590    0.00030   -0.00899    0.00774
MOLE  5
F1    4    0.777489    1.238161    0.346250    11.00000    0.16804
0.09730 =
         0.26003    0.07246   -0.13260   -0.03860
F2    4    0.453873    1.260563    0.302142    11.00000    0.25401
0.16028 =
         0.35001    0.07884   -0.25609   -0.11641
F3    4    0.518441    1.293069    0.426776    11.00000    0.18890
0.67372 =
         0.08178    0.15697    0.01132   -0.07643
F5    4    0.716167    1.366427    0.286762    11.00000    0.13531
0.26025 =
         0.08495    0.09574    0.04358    0.04959
F4    4    0.779979    1.399235    0.411490    11.00000    0.10181
0.09396 =
         0.17525   -0.04943   -0.04301    0.00806
```

```
F6    4    0.457801    1.422728    0.368229    11.00000    0.13009
0.10274 =
         0.59708   -0.11010   -0.12754    0.05227
SB1   7    0.612102    1.331315    0.356893    11.00000    0.07391
0.08823 =
         0.05590    0.01404   -0.01726   -0.01398
MOLE  6
F7    4    1.026333    0.610709    0.128515    11.00000    0.07059
0.04833 =
         0.36353    0.02481    0.04949    0.01627
F8    4    0.694914    0.630716    0.093076    11.00000    0.08104
0.08143 =
         0.12993   -0.00879   -0.04055   -0.02484
F9    4    0.934028    0.752103    0.087656    11.00000    0.10402
0.05430 =
         0.07611    0.00871    0.01860   -0.00026
F10   4    1.010267    0.762475    0.210227    11.00000    0.14098
0.14342 =
         0.06431    0.04929   -0.03644   -0.04139
F12   4    0.679179    0.780804    0.174933    11.00000    0.11893
0.08224 =
         0.13433    0.03001    0.08409    0.02650
F11   4    0.771641    0.638509    0.215207    11.00000    0.19577
0.13819 =
         0.14799    0.09890   -0.04919   -0.05684
SB2   7    0.858099    0.694859    0.151615    11.00000    0.05850
0.04931 =
         0.05641    0.01610   -0.00877   -0.00699
MOLE  7
F13   4    0.274916    0.618325    0.593947    11.00000    0.07783
0.09001 =
         0.16851   -0.05265    0.06299   -0.01079
F14   4   -0.056882    0.599632    0.625992    11.00000    0.06136
0.10843 =
         0.96173   -0.12823    0.04272   -0.01290
F17   4    0.193119    0.629377    0.715514    11.00000    0.38992
0.33213 =
         0.29056    0.27608    0.14417    0.20816
F18   4   -0.044344    0.750551    0.707057    11.00000    0.39937
0.15360 =
         0.30551    0.09826    0.31265    0.15340
F16   4    0.288609    0.770190    0.674118    11.00000    0.16918
0.17658 =
         0.18923    0.01037   -0.08658   -0.02610
F15   4    0.040196    0.741331    0.585595    11.00000    0.22725
0.08563 =
         0.13602    0.04013   -0.03365   -0.00447
SB3   7    0.112744    0.681595    0.652148    11.00000    0.07403
0.08443 =
         0.05580    0.01346    0.01711    0.01730
MOLE  8
F19   4    0.180493    0.228436    0.835963    11.00000    0.08171
0.04355 =
         0.15202    0.03092   -0.02035   -0.01417
F20   4    0.442214    0.260326    0.919767    11.00000    0.09614
0.08863 =
         0.06204    0.00962   -0.02060    0.02965
```

```
F21   4    0.506935    0.246393    0.796050    11.00000    0.10428
0.11643 =
         0.10601    0.03140    0.03722    0.04456
F22   4    0.263055    0.365682    0.789230    11.00000    0.11677
0.14378 =
         0.07707    0.06024   -0.02434   -0.01612
F24   4    0.526610    0.400149    0.873374    11.00000    0.08106
0.07658 =
         0.23291    0.01137   -0.04463   -0.04831
F23   4    0.200036    0.381523    0.913056    11.00000    0.16475
0.09746 =
         0.10397    0.02742    0.01855    0.03939
SB4   7    0.359702    0.316832    0.856753    11.00000    0.05600
0.04420 =
         0.05365    0.01434    0.00679    0.00598
HKLF    4  1.0  1.00  0.00  0.00  0.00  1.00  0.00  0.00  0.00
1.00




REM  mo_VPX186_II_0sa_a.res in P1
REM wR2 = 0.2091, GooF = S = 1.051, Restrained GooF = 1.055 for all
data
REM R1 = 0.0869 for 16935 Fo > 4sig(Fo) and 0.0926 for all 18298
data
REM 608 parameters refined using 111 restraints

END

WGHT      0.0777     30.7115

REM Highest difference peak  4.406,  deepest hole -2.564,  1-sigma
level  0.205
Q1    1   0.9326  0.6639  0.1315  11.00000  0.05    4.41
Q2    1   0.4320  0.3498  0.8784  11.00000  0.05    3.87
Q3    1   0.0237  0.6617  0.6315  11.00000  0.05    2.98
Q4    1   0.5293  1.3519  0.3775  11.00000  0.05    2.78
Q5    1   0.4889 -0.2225  0.9462  11.00000  0.05    2.42
Q6    1   0.9836  1.2379  0.0639  11.00000  0.05    2.40
Q7    1   0.5544  0.8001  0.4090  11.00000  0.05    2.33
Q8    1   0.0522  0.2150  0.6005  11.00000  0.05    1.90
Q9    1   0.6084  1.3147  0.3446  11.00000  0.05    1.85
Q10   1   0.2738  0.3467  0.8566  11.00000  0.05    1.83
Q11   1   0.4805  0.7837  0.4404  11.00000  0.05    1.76
Q12   1   0.0995  0.7001  0.6641  11.00000  0.05    1.76
Q13   1   0.7740  0.6715  0.1549  11.00000  0.05    1.75
Q14   1   0.5993  1.3806  0.3544  11.00000  0.05    1.73
Q15   1   0.6768  0.6839  0.4310  11.00000  0.05    1.72
Q16   1  -0.0440  0.2247  0.5685  11.00000  0.05    1.59
Q17   1  -0.3082  0.3429  0.5846  11.00000  0.05    1.57
Q18   1   0.1037  0.6367  0.6549  11.00000  0.05    1.56
Q19   1   0.0047  0.1070  0.6328  11.00000  0.05    1.51
Q20   1  -0.2488  0.3295  0.6423  11.00000  0.05    1.50
```

4. Coordinates of calculated structures of 3 and 3’

3

C	0.609900	-2.780400	-0.314600
C	1.832100	-2.260300	0.193700
C	1.845200	-1.432300	1.368400
C	0.617800	-1.164200	2.048400
C	-0.625200	-1.680400	1.549500
C	-0.616500	-2.483900	0.372000
C	0.585400	-3.603300	-1.569900
C	3.129900	-0.838100	1.874600
C	-1.912000	-1.349500	2.252300
H	1.483700	-3.430200	-2.182400
H	0.550600	-4.674200	-1.295400
H	-0.306200	-3.374200	-2.174100
H	-1.563000	-2.837300	-0.047300
H	-2.776900	-1.432300	1.577100
H	-2.058600	-2.061300	3.085600
H	-1.886100	-0.334200	2.678500
H	0.618900	-0.510400	2.925700
H	3.875100	-0.732600	1.071100
H	2.959300	0.148000	2.335900
H	3.556500	-1.506200	2.645500
H	2.763500	-2.441600	-0.351100
C	1.213200	2.245000	0.275300
C	2.000700	3.401400	0.252000
C	3.255500	3.363800	-0.369800
C	3.681700	2.170900	-0.971400
C	2.850600	1.051200	-0.911900
C	-0.161900	2.167400	0.892300

C	-2.178400	1.105800	0.066200
C	-4.063100	0.020500	-1.014200
C	-4.971400	0.869900	-0.420800
C	-4.463700	1.897200	0.423700
C	-3.108400	2.026400	0.661300
N	1.656200	1.089400	-0.282300
N	-0.867700	1.066000	0.260000
N	-2.732100	0.153300	-0.772700
H	-2.050400	-0.393000	-1.322500
H	-4.341300	-0.784700	-1.700700
H	-6.041700	0.754000	-0.606900
H	-5.159600	2.591000	0.909100
H	-2.735400	2.798200	1.337500
H	-0.676300	3.134900	0.752000
H	-0.061400	2.009600	1.989000
H	1.623400	4.320100	0.711800
H	3.886100	4.258700	-0.397800
H	4.644400	2.101600	-1.486600
H	3.125200	0.097700	-1.371800
Cl	-0.060200	-0.343600	-2.424200
Ru	0.410700	-0.554200	-0.051100

3'

C	-2.000200	-1.886500	1.087900
C	-2.744800	-0.721300	0.743000
C	-2.771300	-0.226800	-0.609300
C	-2.042400	-0.930600	-1.613700
C	-1.254600	-2.083500	-1.277900
C	-1.241800	-2.551100	0.068400
C	-1.967900	-2.385900	2.502100

C	-3.530100	1.023400	-0.954200
C	-0.420600	-2.773300	-2.323000
H	-2.204400	-1.584100	3.218100
H	-2.726300	-3.184300	2.609800
H	-0.983100	-2.808900	2.752100
H	-0.594900	-3.390200	0.340000
H	0.554100	-3.084000	-1.911500
H	-0.948900	-3.681400	-2.666500
H	-0.258600	-2.133800	-3.206700
H	-2.025800	-0.545100	-2.637900
H	-3.616800	1.697000	-0.087300
H	-3.048500	1.567300	-1.782600
H	-4.553400	0.750100	-1.271500
H	-3.268400	-0.168500	1.529100
C	0.273200	2.253600	-0.841800
C	0.474300	3.637300	-0.872900
C	-0.144200	4.439200	0.095400
C	-0.928300	3.827100	1.082500
C	-1.078300	2.438900	1.063500
C	0.869400	1.301900	-1.840600
C	2.382800	-0.245500	-0.585200
C	3.972900	-1.824400	-0.092700
C	4.773600	-0.884400	0.572000
C	4.322200	0.441500	0.638800
C	3.096600	0.779500	0.052500
N	-0.505100	1.676000	0.107700
N	1.098800	-0.025900	-1.210900
N	2.801900	-1.509100	-0.667500
H	4.290000	-2.873300	-0.173000
H	5.724400	-1.185700	1.023400

H	4.913300	1.209200	1.150000
H	2.709900	1.799600	0.110200
H	1.799000	1.703400	-2.277700
H	0.149600	1.147600	-2.663300
H	1.114300	4.071700	-1.646700
H	-0.003400	5.525100	0.087000
H	-1.414900	4.410100	1.869900
H	-1.655600	1.909000	1.825700
H	1.027900	-0.757100	-1.921900
Cl	0.663600	-0.325500	2.035700
Ru	-0.688000	-0.400500	0.048900

5 References

(1) (a) Race, J. J.; Albrecht, M. Pyridylidene Amines and Amides: Donor-Flexible Ligands for Catalysis. *ACS Catal.* **2023**, *13* (14), 9891–9904. https://doi.org/10.1021/acscatal.3c02203. (b) Shi, Q.; Thatcher, R. J.; Slattery, J.; Sauari, P. S.; Whitwood, A. C.; McGowan, P. C.; Douthwaite, R. E. Synthesis, Coordination Chemistry and Bonding of Strong N-Donor Ligands Incorporating the 1*H*-Pyridin-(2*E*)-Ylidene (PYE) Motif. *Chemistry A European J* **2009**, *15* (42), 11346–11360. https://doi.org/10.1002/chem.200901382.

(2) Sheldrick, G. M. Crystal Structure Refinement with *SHELXL*. *Acta Crystallogr C Struct Chem* **2015**, *71* (1), 3–8. https://doi.org/10.1107/S2053229614024218.

(3) Farrugia, L. J. *WinGX* and *ORTEP for Windows* : An Update. *J Appl Crystallogr* **2012**, *45* (4), 849–854. https://doi.org/10.1107/S0021889812029111.
